# A Review of 3D Polymeric Scaffolds for Bone Tissue Engineering: Principles, Fabrication Techniques, Immunomodulatory Roles, and Challenges

**DOI:** 10.3390/bioengineering10020204

**Published:** 2023-02-03

**Authors:** Ahmed G. Abdelaziz, Hassan Nageh, Sara M. Abdo, Mohga S. Abdalla, Asmaa A. Amer, Abdalla Abdal-hay, Ahmed Barhoum

**Affiliations:** 1 Biochemistry Division, Chemistry Department, Faculty of Science, Helwan University, Cairo 11795, Egypt; 2 Nanotechnology Research Centre (NTRC), The British University in Egypt, Cairo 11837, Egypt; 3 Department of Pharmacognosy, Pharmaceutical and Drug Industries Research Institute, National Research Centre, Giza 12622, Egypt; 4 Department of Mechanical Engineering, Faculty of Engineering, South Valley University, Qena 83523, Egypt; 5 Faculty of Industry and Energy Technology, Mechatronics Technology Program, New Cairo Technological University, Cairo 11835, Egypt; 6 School of Chemical Sciences, Dublin City University, D09 Y074 Dublin, Ireland

**Keywords:** tissue engineering and regenerative medicine, biopolymers, nanofabrication techniques, additive manufacturing, rapid prototyping, customized therapy 3D scaffolds

## Abstract

Over the last few years, biopolymers have attracted great interest in tissue engineering and regenerative medicine due to the great diversity of their chemical, mechanical, and physical properties for the fabrication of 3D scaffolds. This review is devoted to recent advances in synthetic and natural polymeric 3D scaffolds for bone tissue engineering (BTE) and regenerative therapies. The review comprehensively discusses the implications of biological macromolecules, structure, and composition of polymeric scaffolds used in BTE. Various approaches to fabricating 3D BTE scaffolds are discussed, including solvent casting and particle leaching, freeze-drying, thermally induced phase separation, gas foaming, electrospinning, and sol–gel techniques. Rapid prototyping technologies such as stereolithography, fused deposition modeling, selective laser sintering, and 3D bioprinting are also covered. The immunomodulatory roles of polymeric scaffolds utilized for BTE applications are discussed. In addition, the features and challenges of 3D polymer scaffolds fabricated using advanced additive manufacturing technologies (rapid prototyping) are addressed and compared to conventional subtractive manufacturing techniques. Finally, the challenges of applying scaffold-based BTE treatments in practice are discussed in-depth.

## 1. Introduction

Tissue engineering (TE) is a discipline of biomedical engineering that uses a combination of cells, technology, material methods, and appropriate biochemical and physicochemical factors to restore, maintain, enhance, or replace various types of biological tissue. The terminology TE was first introduced to the scientific community in 1987 [1]. It can be described as a multidisciplinary approach aimed at replacing damaged biological tissue. As a result of rapidly developing technologies, bone tissue engineering (BTE) has emerged as a promising approach to reconstructing large segmental bone defects. Scaffolds, the key component of tissue engineering, are designed to simulate host tissue functions and provide a suitable microenvironment for the proliferation and differentiation of host cells and the reconstruction of new healthy tissue. To design an ideal scaffold for the tissue engineering of bone, it must have a number of crucial properties, such as biodegradability, biocompatibility, osteoinductivity, osteoconductivity, bioactivity, and various other surface properties such as suitable porosity and surface roughness [2]. The replacement of damaged tissues with an artificial prosthesis goes back to the past when archeologists excavated materials such as metals (gold and silver), shells, and corals that were used to replace broken/missing human bones. For example, the Etruscans replaced damaged teeth with ox bones in the 6th century BC [3].

Since bone tissue damage occasionally occurs due to accidental trauma and pathological causes, more than 2.2 million bone grafts are performed annually worldwide [4,5]. Currently, treatment protocols for bone tissue damage primarily focus on autologous and allogeneic grafts, with autologous grafts considered the gold standard [5]. However, using bone grafts to treat bone tissue damage is associated with several limitations, including the risk of developing an immune response, an inadequate supply of grafts, donor site morbidity, and the need for additional procedures [6]. Regenerative medicine and tissue engineering have emerged in recent decades as promising approaches for the repair of bone tissue damage, with the goal of reducing the complications associated with conventional methods [7,8,9,10]. Biomaterials for bone tissue engineering (BTE) can be described as impermanent matrices that provide a suitable microenvironment for cell proliferation and differentiation. Scaffolds, on the other hand, are considered model structures that support three-dimensional (3D) tissue reconstruction [9,11]. Scaffolds are used either as cell-free microenvironments or as carriers for cells or/and drugs. Cell-free scaffolds must allow the settlement of host cells once implanted at the injury site for the regeneration process to occur. The scaffolds, combined with various cell types of different lineages, can trigger bone formation in vivo through osteogenic differentiation or the release of soluble mediators. Researchers have used the most common cells for this purpose: adult stem cells (stem cells derived from bone marrow, adipose tissue, and peripheral blood), embryonic stem cells and induced pluripotent stem cells, and genetically modified cells [7,9].

Nowadays, the most common fabrication technologies for processing biomaterials into 3D scaffolds for tissue engineering are (i) conventional and (ii) rapid prototyping approaches [12]. Examples of conventional techniques include solvent casting and particle leaching [13], freeze-drying [14], thermally induced phase separation (TIPS) [15], gas foaming [16], electrospinning [17], and sol–gel techniques [18]. However, the application of conventional techniques has its limitations, such as the high cytotoxicity of organic solvents and the difficult control of scaffold microstructure and accuracy [19]. Rapid prototyping (RP) technologies are more commonly known as additive manufacturing, as the material is applied layer by layer in a stepwise manner until the final shape is achieved. Rapid prototyping technologies include stereolithography [20], fused deposition molding [21], selective laser sintering (SLS) [22,23], and 3D bioprinting [24]. These rapid prototyping technologies eliminate the need for cytotoxic organic solvents used in conventional methods [25]. The pore size and neat geometry can be precisely controlled. Currently, the use of scaffolds for BTE still faces many obstacles. These include finding suitable materials for scaffold fabrication, the high cost of in vitro, in vivo, and clinical studies, marketing the new scaffold-based products, convincing patients to try new scaffold-based treatments, and trying to meet their high expectations. Finally, the complex regulations governing the use of biomedical devices vary immensely from country to country [21].

This review aims to spotlight the potential applications of biopolymers and their 3D scaffolds in BTE and to discuss the current challenges of both natural and synthetic polymers. The hierarchical structure and chemical composition of bone tissue is highlighted. Different phases of secondary bone fracture healing are discussed. In addition, the essential requirements for the fabrication of ideal BTE scaffolds are discussed in detail. Conventional approaches for fabricating 3D BTE scaffolds are discussed including solvent casting and particle leaching, freeze-drying, thermally induced phase separation, gas foaming, electrospinning, and sol–gel techniques. Rapid prototyping technologies such as stereolithography, fused deposition molding, selective laser sintering, and 3D bioprinting are also discussed. The advantages and disadvantages of 3D BTE scaffolds fabricated using different fabrication technologies are discussed in detail. Finally, the challenges in the application of these novel BTE therapies and their future prospects are discussed.

## 2. Bone Composition and Structure

**Figure 1:**
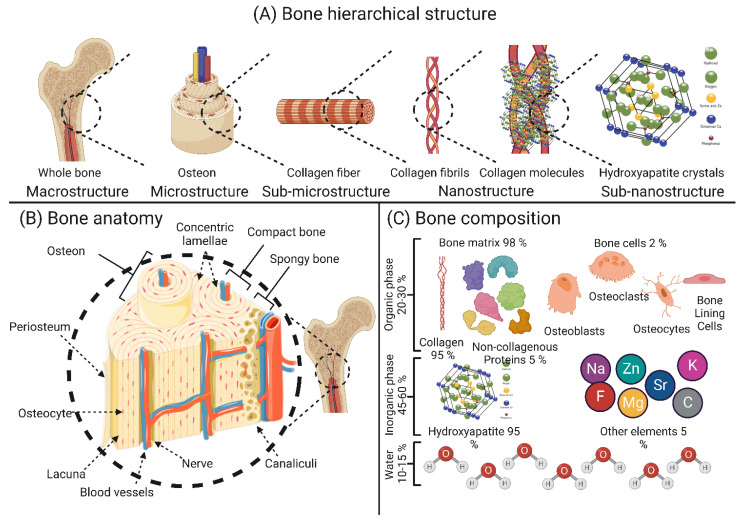
Schematic representation of the basic structure of bone tissue: (**A**) hierarchical structure of the bone; (**B**) anatomical features of the bone; (**C**) elemental composition of the bone. The image was created using Biorender.

Bone tissue is considered a special type of connective tissue with mineral inclusions, which performs many important functions in the body of the organism: locomotion, protection and support of soft tissue weight, the main calcium and phosphate storage in the body, and a reservoir for bone marrow [26]. From a chemical point of view, bone tissue is a composite material consisting of 45–60% (weight/weight) inorganic minerals, 20–30% (weight/weight) organic materials, and 10–20% (weight/weight) water. Most of the organic matrix of bone is composed of type I collagen, which has an aligned triple helix structure [27]. The remaining smaller portion of the bone matrix consists of non-collagenous proteins (NCPs), which are composed of non-collagenous glycoproteins and bone-specific proteoglycans. Some examples of these proteins are osteocalcin, osteonectin, bone sialoproteins, bone phosphoproteins, and small proteoglycans [28]. These non-collagenous proteins play an important role in the mineralization of bone and the association of cells and matrix with structural proteins. Growth factors, secreted by bone cells and having an effect on bone cells themselves, account for less than 1% of non-collagenous proteins [29]. The inorganic part of bone tissue is mainly composed of hydroxyapatite (HA) with the chemical formula Ca_10_(PO_4_)_6_(OH)_2_. The composition of HA crystals changes with time, so their biological properties depend on the amount and age of the crystallites [30]. The chemical composition and anatomy of bone are depicted in Figure 1.

Through the activity of highly specialized cells, namely osteoblasts, osteoclasts, osteocytes, and bone lining cells, bone remodeling occurs constantly throughout a person’s life to ensure a balance between the process of bone resorption and new bone formation [31]. Osteoblasts are best known as bone-forming cells and account for 4% to 6% of the total bone cell [32]. Osteoblasts are responsible for the synthesis of various bone proteins involved in bone hemostasis, such as type I collagen, osteonectin, osteopontin (OPN), γ-carboxy proteins, osteocalcin (OCN), proteoglycans, and alkaline phosphatase (ALP). Numerous growth factors are also synthesized by osteoblasts, such as transforming growth factor β (TGF-β), insulin-like growth factors I and II (IGF-I and IGF-II), and bone morphogenetic proteins (BMPs) [33]. While osteocytes settle on the bone surface where little or no bone resorption/formation occurs [34], bone lining cells regulate the influx and efflux of minerals at sites where the bone is in contact with other tissues [35]. Osteocytes are the most abundant bone cells and account for 90–95% of the bone cell population. These cells perform several important functions in hemostasis in bone, such as (i) harmonization of osteoblast and osteoclast activity, (ii) endocrine regulation of phosphate balance, (iii) sensors of local mechanical stress, and (iv) regulation of cell signaling [36]. Osteoclasts play an essential role in the initial phase of bone remodeling (i.e., resorption), as they can engulf aged/damaged bone matrix to make room for the synthesis of neo-bone tissue [31].

The macroscopic structure of bone is based on the repetitive arrangement of microscale units. These microscale units are assembled in nanoscale structures [37,38]. At the macroscale, bone is divided into two categories: cortical (compact) and cancellous (trabecular) bone. The macrostructure of the long limb bones (e.g., femur, tibia, and fibula) shows an inner trabecular bone surrounded by an outer compact bone, whereas the macrostructure of the flat bones (e.g., skullcap) shows more of a sandwich arrangement [29,30]. Microscopically, the collagen fibers with mineral intercalations are arranged in planar structures called lamellae, which are 3–7 µm wide. These lamellae are arranged concentrically around a central channel (Haversian channel) to form what are known as osteons [39]. Osteons are concentric rings 200–250 µm in diameter that runs parallel to the bone’s long axis [40]. At the nanoscale structure, bone is composed mainly of mineral-deposited collagen fibrils. These collagen fibrils and mineral crystals, whose size is in the range of tens of nanometers (nm), are composed of sub-nano mineral crystals, collagen molecules, and molecules of non-collagenous proteins [40]. The hierarchical structure and composition of the bone are shown in Figure 1.

## 3. Bone Reconstruction and Self-Healing Capacity

Loss of bone tissue may be due to various causes, including surgical removal of bone tumors (osteosarcomas), diseases affecting bone quality, bone infections (osteomyelitis), and traumatic injuries. Currently, the above bone damage/losses are treated with autologous grafts (gold standard), allogeneic grafts, and metal prostheses [40,41]. (1) Bone infection (osteomyelitis): osteomyelitis stands for bone marrow inflammation [42]. Osteomyelitis may originate from a single area or from multiple areas, including the cortex, periosteum, and bone marrow, as well as the soft tissues surrounding the affected area [43]. (2) Diseases affecting the quality of bone: in general, bone undergoes continuous remodeling/reconstruction in a dual process of bone resorption and bone formation. Bone remodeling allows the bone to remove damaged parts and replace these damaged parts with new bone, thereby increasing bone strength. However, if bone resorption and bone formation are not evenly balanced, this can result in a net gain or loss of bone tissue. Therefore, bone remodeling/turnover affects bone quality and bone mineral density (BMD) [44,45]. (3) Bone cancer (osteosarcoma): osteosarcoma is the most common primary malignant tumor of bone tissue in clinical practice. It arises from mesenchymal tissue composed of stromal cells with spindle-shaped morphology that can form bone-like tissue. Osteosarcomas account for approximately 20% of all cases of primary malignant tumors registered worldwide [46,47].

**Figure 2:**
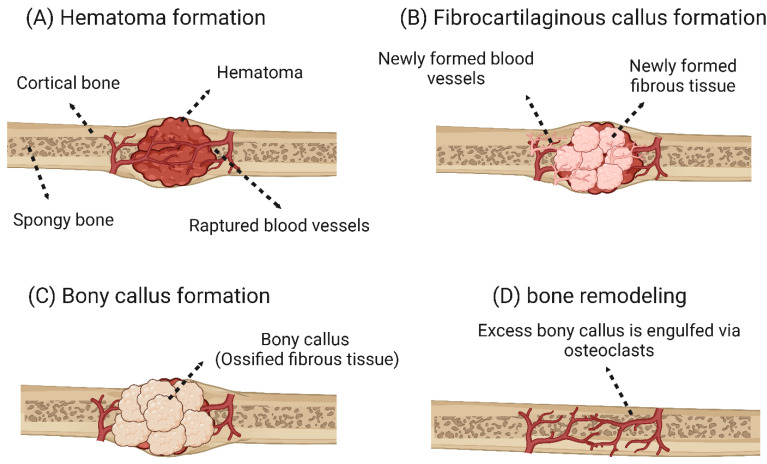
Main stages of secondary bone healing. (**A**) A hematoma forms after the fracture. (**B**) In the first stage of regeneration, the fibrin is gradually replaced by fibrous-cartilaginous tissue forming woven bone. (**C**) At a later stage of the regeneration phase, ossification of the cartilaginous tissue occurs, and more neocartilaginous tissue is formed. (**D**) Once the bone has grown back together over bony calluses, the original morphology of the bone cortex is restored by remodeling [48]. The image was created using Biorender.

Bone reconstruction and self-healing: fracture reconstruction is a reconstruction process that can be divided into two main types: primary and secondary bone healing. Primary bone healing occurs in cases where bone fragments are held tightly together under pressure. This type of bone healing is characterized by the absence of bony calluses and the rejoining of the ends of the two bone fragments by the action of osteoblasts and osteoclasts. Thus, the healing process takes place directly [31,32]. Secondary bone healing is the most common form of bone healing and occurs in response to low mobility at the fracture site, leading to the formation of soft callus and then secondary bone formation by ossification [31,33]. Secondary bone healing is divided into four main stages: (i) hematoma formation at the fracture site: immediately after the fracture, a hematoma forms as a result of the death of bone cells and swelling of the tissue at the fracture site (this phase is characterized by the development of a new blood supply that grows into the hematoma, and phagocytic cells begin to engulf and digest debris). This process is also characterized by the immigration of osteoblasts and osteoclasts to the fracture site. (iii) Bone callus formation: this process also occurs three to four weeks after fracture. In this phase, there is a proliferation of osteoblasts and osteoclasts, the action of which transforms the fibrous-cartilaginous calluses into bony calluses. (iv) Remodeling of the bone: in this stage, excess bone calluses are removed, and cortical bone is built up to reconstruct the bone diaphysis [34,35]. The four main stages of secondary bone healing are shown in Figure 2.

## 4. Properties of Ideal Polymeric Scaffolds for Bone Tissue Engineering

To construct an ideal BTE framework, some properties must be present within that framework.

**Figure 3:**
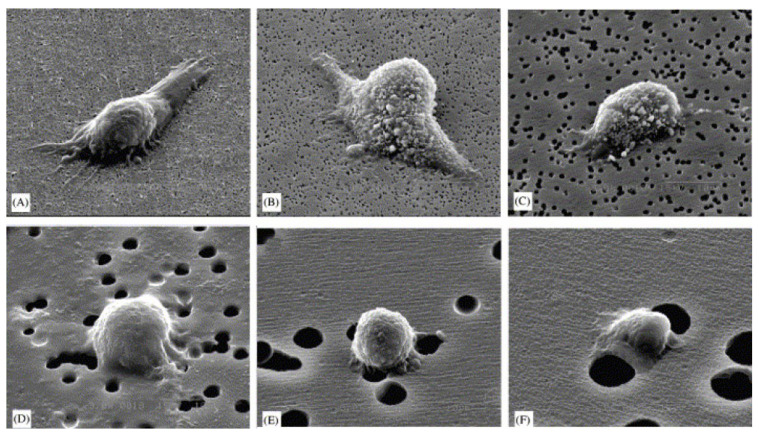
SEM images of adhesion of MG63 cells to the PC membrane surfaces with different surface roughnesses: (**A**) 0.2, (**B**) 0.4, (**C**) 1.0, (**D**) 3.0, (**E**) 5.0, and (**F**) 8.0 mm [78]. © Elsevier, 2004.

**Figure 4:**
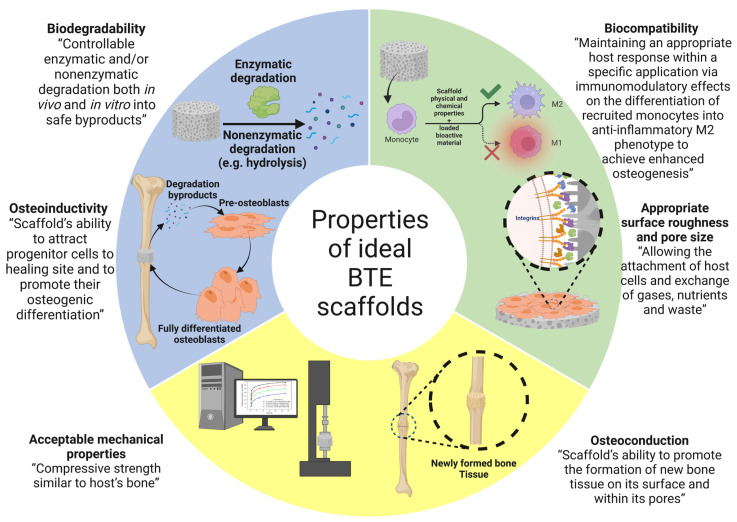
The properties of ideal bone tissue engineering scaffold. The image was created using Biorender.

(1) Biocompatibility: the biocompatibility of biomaterials or biomedical devices can be defined as their ability to perform their functions while maintaining an appropriate host response within a specific application. It can be studied by measuring the extent of adverse changes that affect homeostasis and thus determines the host response to the implanted biomaterial/biomedical device [49]. Therefore, the application in which the biomaterial is used should be specified as biocompatible or non-biocompatible [50]. In 1963, Charnley invented a metal-on-plastic hip replacement made of polytetrafluoroethylene (PTFE) [51]. Many years after Charnley’s findings, PTFE-based monolithic materials were considered biocompatible and were used clinically after passing all preclinical biosafety studies [50]. As reported by Williams [50], the biological host environment should be considered because the interaction between implanted biomaterials and host tissue is time-dependent and the biomaterial itself may undergo conditioning after a period of contact with the host tissue. Biocompatibility is a critical property that BTE scaffolds must possess. Biocompatible BTE scaffolds should: (i) allow cell-to-cell communication via biomolecular signaling while being non-toxic to the surrounding host tissue [52]; (ii) have some degree of osteoconductivity, which refers to the ability of the scaffold to allow the synthesis of new bone tissue on its surface and within its pores [53]; (iii) exhibit some degree of osteoinductivity, which refers to the ability of the scaffold to recruit progenitor cells to the healing site and promote their osteogenic differentiation [53]; (iv) induce neovascularization to allow the exchange of nutrients, waste products, and oxygen through the newly formed blood vessels surrounding the implant [54]. However, implantation of polymer scaffolds often results in the initiation of an inflammatory response leading to the recruitment of immune cells, particularly monocytes, which differentiate into either macrophages of the inflammatory M1 or anti-inflammatory M2 phenotype; a biocompatible scaffold should promote the differentiation of monocytes into the anti-inflammatory M2 macrophage phenotype [55]. Thus, the immunomodulatory effect of the scaffold can be tailored to enhance bone regeneration. The immunomodulatory role of polymer scaffolds is discussed in detail in the following section.

(2) Biodegradability: this refers to the ability of the biomaterial to be actively degraded by enzymatic action or passively degraded by hydrolysis both in vitro and in vivo [56]. It should be noted that the mechanism by which polymer-based biomaterials are degraded depends primarily on the type of bonds found in their backbone. For example, polycarbonates with hydrolytically very stable bonds such as carbonyl and ether linkages cannot be passively degraded by hydrolysis and require the assistance of enzymes to be degraded at a reasonable rate [57]. Other polymers that have bonds such as esters, ortho-esters, amides, anhydrides, and phosphates can be passively degraded by hydrolysis under physiological conditions [58]. Biodegradability is a key property that should be considered in the design of BTE scaffolds [59]. A successful BTE scaffold must be biodegradable in vivo at a controlled rate and release non-cytotoxic byproducts [60]. However, different applications require implants with different degradation rates. For example, in the case of severe bone tissue damage, the implant may be permanent [61]. Spinal fusion requires an implant that degraded completely after nine months. For craniomaxillofacial applications, the ideal implant should be completely degraded after three to six months [62]. As Dorozhkin reported, the overall architecture of the scaffold changed with degradation, and the released byproducts affected the osteoinductivity and osteoconductivity of the scaffold [3].

(3) Mechanical properties: for a BTE scaffold to effectively replace defective bone tissue, the scaffold should have similar mechanical properties to the host bone at the defect site, and it must accelerate bone healing after implantation [63,64]. It should be noted that the mechanical properties of human bone vary drastically depending on the bone type. According to the results of Olszta et al. [65], compact bone had a modulus of elasticity of 15–20 GPa, while this value was much lower for trabecular bone, 0.1–2 GPa. On the other hand, the compressive strength for compact bone was 100–200 MPa, while much lower values between 2 and 20 MPa were observed for trabecular bone. Due to these drastic differences in the mechanical properties of bone, the fabrication of an “optimal scaffold” for BTE is a complicated process, as the mechanical properties of the host bone tissue and the implant should be very similar. Another important characteristic of the mechanical strength of the scaffolds is their fatigue behavior under stress. Fatigue failure of polymeric scaffolds is due to the repetition of stresses less than the ultimate compressive strength of the scaffold, which eventually leads to cracks as a result of these repetitive load cycles. Fatigue failure can be caused either thermally by melting the polymer material or mechanically by repetitive stress/strain cycles. Polymeric scaffolds exhibit fatigue failure similar to metallic prostheses in that it begins with microscopic cracks that eventually grow to macroscopic cracks and eventually lead to scaffold failure. Depending on the type of strain, fatigue testers include (i) an axial loading testing apparatus, which applies cycles of uniform compression or tension scaffolds uniformly until the scaffold network fails; (ii) rotational bending testers, in which the scaffold is cyclically bent and compressed; (3) fracture testing devices in which the initiation and propagation of a fracture are tested by placing a notch in the framework with repeated cycles of compression.

(4) Pore size/porosity: the ability of the scaffold to promote osteogenesis on its surface and in its pores (osteoconductivity) depends on its microporosity, which mainly depends on pore size, volume, and interconnectivity. The porosity of the scaffold is a crucial factor for the influx of oxygen and nutrients and the efflux of waste products, which are essential for cell growth, migration, and proliferation, as well as for providing a suitable structure for the synthesis and neovascularization of the ECM [66]. Based on their average pore size, scaffolds can be classified into three main types: (i) macroporous scaffolds with an average pore size greater than 100 µm, (ii) microporous scaffolds with an average pore size less than 10 µm, and (iii) mesoporous scaffolds with an average pore size less than 100 nm [67]. However, the optimal porosity and pore size required to build a three-dimensional BTE scaffold have not been clearly elucidated [68]. However, Karageorgiou and Kaplan reported that scaffolds with a pore size of less than 200 µm supported osteoblast survival both in vivo and in vitro, and osteogenesis was restricted to the surface and periphery of the scaffolds due to the lack of oxygen and nutrient flow in the scaffolds [69]. Karageorgiou and Kaplan also showed that scaffolds with pores with a mean diameter of 300 µm promoted the proliferation and differentiation of osteoblasts within the entire scaffold due to enhanced oxygen and nutrient diffusion and angiogenesis [69]. It has been reported that a pore size of 200 to 350 µm allows optimal osteogenesis and that scaffolds with pores support better bone formation at both micro- and macroscale compared to scaffolds with only macropores [70].

(5) Surface roughness/topography: the surface roughness of the scaffold directly affects the ability of host cells to attach [71,72] and it also directs host response and modulates the crosstalk between different cell components [73]. Macroscopically, the implant should be fixed at the implantation site. Microscopically, the cells interact directly with the micro- and sub-microstructures of the implant. At the nanoscale, cells have been observed to interact with integrin binding sites of the implant through their receptors [74,75]. Different cell types seem to preferentially adhere to different surface topographies. For example, fibroblasts have been shown to adhere better to smooth surfaces, while they are stimulated to proliferate and synthesize collagen when seeded on surfaces with intermediate roughness. In contrast to fibroblasts, epithelial cells should adhere better to rough surfaces. In addition, the nanoscale topographic features of the scaffold were found to influence ECM synthesis, adhesion, proliferation, and differentiation of osteoblasts [76,77]. In a study conducted by Lee and his colleagues [78], it was found that MG-63 osteosarcoma cells seeded on polycarbonate membranes (PC) with different surface roughness (200 nm to 8 µm) behaved differently (Figure 3). With increasing surface roughness, there was a gradual inhibition of cell adhesion and proliferation. Lee et al. [78] explained that this inhibition was due to the large discontinuities on the surface.

(6) Osteoconductivity: osteoconductivity refers to the ability of the scaffold to promote osteogenesis on its surfaces and pores, as it should allow proliferation and adhesion of bone-forming cells as well as ECM formation on its entire surfaces [79]. The osteoconductivity of the scaffold is determined by several parameters such as chemical composition, architecture, biodegradability, biocompatibility, hydrophilicity, porosity, and mechanical properties of the scaffold. An ideal scaffold should have a mean pore size of 100 µm to allow angiogenesis as well as the diffusion of nutrients, waste, and oxygen required for osteogenesis [80].

(7) Osteoinduction: osteoinduction can be described as the ability of the scaffold to recruit progenitor cells to the healing site and promote their osteogenic differentiation via biomolecular signaling [77]. It has been observed that the rough surface and nanoscale structures of the scaffold promote the osteogenic differentiation of stem cells into osteoblasts. It has also been observed that implants with reduced oxygen partial pressure promote the dedifferentiation of pericytes in blood vessels into bone-forming cells [79]. The characteristics of an ideal BTE scaffold are shown in Figure 4.

## 5. Polymer Materials Used in Polymer Scaffolds for Bone Tissue Engineering

Polymer scaffolds can be made from both natural and synthetic polymers. The advantages of natural polymers are their exceptional biocompatibility, osteoinductive capabilities, and lower likelihood of eliciting an immune response [81,82,83]. Natural polymers have many advantages in BTE applications, including their high biodegradability, biocompatibility, the presence of cell adhesion sites, high biomimicry due to their similarity to the native ECM, a high degree of bioactivity, and the fact that their processing does not require the use of strong chemicals. However, the use of natural polymers in BTE applications is still associated with many problems, such as their mediocre mechanical properties, the high cost of their fabrication and isolation, their low thermostability, and their susceptibility to cross-contamination [84,85]. There are also some disadvantages associated with the use of natural polymers, such as the fact that their degradation rate cannot be fully controlled, and their mechanical properties are inferior. Natural polymers are generally either proteins or polysaccharides [36]. Protein-based scaffolds exhibit higher cell adhesion compared to polysaccharide-based scaffolds because the amino acids that form the proteins are involved in cell adhesion through integrin-binding domains. The cellular adhesion and osteoconductivity of polysaccharide-based scaffolds can be improved either by chemical modification of their surface, by association with an osteoconductive material, or by combination with cell adhesion proteins [37,39]. The most commonly used natural polymers in BTE are silk [86], chitosan [87], alginate [88], keratin [89], collagen [70], glycosaminoglycans (GAGs) [90], and hyaluronic acid [91]. The advantages and disadvantages of protein- and polysaccharide-based natural polymers in BTE applications are listed in Table 1.

On the other hand, synthetic polymers have several optimistic features for BTE applications, including their high purity, tunable mechanical and chemical properties, long shelf life, ability to be uniformly produced in large quantities, and low cytotoxicity. However, the use of synthetic polymers in BTE applications still poses many challenges, such as their low biodegradability, cytotoxic degradation products, unclear cell–matrix interactions, adverse effects due to prolonged retention in the body (non-degradable polymers), and their low extensibility [92,93]. Synthetic polymers such as PCL, polyetheretherketone (PEEK), poly(glycolic acid) (PGA), polypropylene fumarate (PPF), and polylactic acid (PLA) have higher mechanical properties, manipulable degradation rates, and long shelf life, and can be produced inexpensively on a large scale. However, the use of synthetic polymers has some disadvantages, including their lower bioactivity compared to natural polymers [94]. Synthetic polymers have proven to be extremely advantageous in biomedical applications because they are easy to manufacture, inexpensive, tunable in properties, have superior mechanical properties, and have easily controlled physicochemical and morphological characteristics [92]. However, the use of synthetic polymers in biomedical applications also has some drawbacks, such as their considerable hydrophobic nature, which affects their ability to transport hydrophilic drugs, the striking irregular degradation behavior due to a phenomenon called autocatalysis, and the denaturation of biologically active proteins and inflammation of surrounding tissues due to their acidic degradation products [95]. Some of the most commonly used synthetic polymers in biomedicine are PLA [96], PCL [97], PGA [98], and PLGA [99].

### 5.1. Natural Polymers-Based Composite Scaffolds

**Figure 5:**
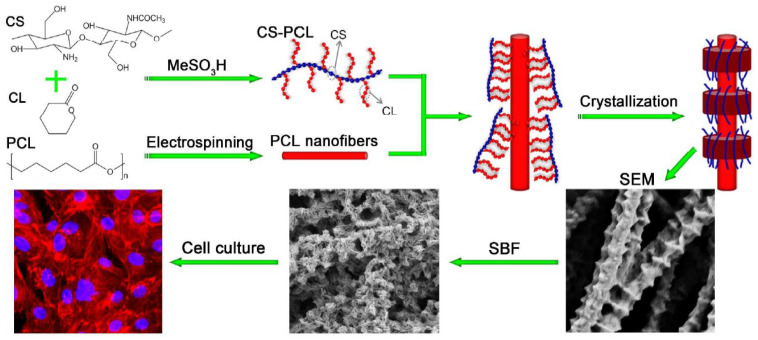
Preparation of chitosan-PCL biocomposite scaffolds displaying “shish kebab-like” morphology [120].

To enhance the properties of natural polymers, they are often combined with other materials such as other polymers (natural or synthetic) or bioceramics (TCP or HAp) to form biocomposites. For example, the poor mechanical properties of pure chitosan nanoscaffolds have paved the way for the production of biocomposites by combining chitosan with other polymers and nano-sized bioactive particles. To overcome its poor mechanical properties, chitosan has been blended with various synthetic polymers such as polymethyl methacrylate (PMMA) [118], PEG [119], PCL [120], and PLA [121]. Since most scaffolds synthesized from synthetic polymers lack cell recognition sites and cell affinity and have low hydrophilicity, natural–synthetic polymer blends are becoming increasingly popular in this research area [121]. X Jing et al. [120] reported the preparation of a chitosan-PCL composite with a unique “shish kebab-like” morphology. This composite was prepared by crystallizing chitosan-PCL copolymers “kebabs” on the surface of electrospun PCL nanofibers (“shish”) Figure 5. This resulted in higher surface roughness of the nanofibers, improving cell adhesion, and integrin binding sites were created by the chitosan-PCL structures (“kebabs”), leading to an increase in cell viability and proliferation.

Chitosan biocomposites have good biocompatibility and osteoconductivity, but they may still lack the required osteoinductivity. The two most commonly used approaches to improving the osteoinductive properties of chitosan biocomposites are either doping with trace elements or incorporating cytokines into biocomposite scaffolds [122]. The most commonly used cytokines for this approach are platelet-derived growth factor (PDGF), vascular endothelial growth factor (VEGF), and bone morphogenetic protein-2 (BMP-2) [123]. However, the major problem with this approach is that the release of cytokines is difficult to control because it depends on the microstructure of the scaffold as well as some other factors such as temperature, pH of the medium, porosity, surface topography, and chemical composition of the scaffold [124]. Tong et al. [125] reported the preparation of a three-dimensional VEGF-silk fibroin-chitosan scaffold (VGEF-SF-CS). The scaffolds were prepared by lyophilization of a premixed solution of SF, CS, and VEGF. The authors reported that an in vitro assay performed with the osteoblast cell line hFOB1.19 using Cell Counting Kit-8 (CCK-8) showed that the scaffolds from VEGF-SF-CS had a higher proliferation rate compared with the scaffolds from SF-CS after 3 days of cultivation. The authors also reported that VEGF-SF-CS scaffolds showed the highest ALP activity after 4–10 days of cultivation. However, it was reported by the authors that the incorporation of VEGF had no significant effect on the adhesion of hFOB1.19 osteoblast cells.

The second approach to improve the osteoconductivity of chitosan biocomposite scaffolds involves doping HAp with trace elements such as Sr^2+^, Zn^2+^, Mg^2+^, Cu^2+^, and Si^4+^ through substitution with Ca^2+^ ions, which can accelerate neovascularization and promote osteogenic differentiation of mesenchymal stem cells (MSCs). Strontium (Sr^2+^) is a promising candidate in BTE because it can stimulate osteogenesis and inhibit the process of bone resorption [126]. Yong et al. [25] reported the preparation of a nanohybrid Sr-HAp chitosan scaffold. They investigated the effect of different concentrations of nano-sized Sr-HAp crystals on the osteoinductivity of chitosan scaffolds. As reported by the authors, the Sr-HAp chitosan scaffold stimulated the mesenchymal stem cells to proliferate and undergo osteogenic differentiation. They also reported that doping HAp with Sr increased ECM mineralization and ALP activity in MSCs and enhanced their expression of ALP and osteogenic COL-1. The synergism between Sr^2+^ and Ca^2+^ in the nanohybrid scaffold developed by the authors showed that Sr is a promising candidate for BTE applications.

Several alginate–chitosan composites have been fabricated and used in BTE applications. Li et al. [127] fabricated alginate–chitosan composites that were tested in vivo for filling and reconstructing bone defects in Sprague Dawley rats. After 16 weeks of filling the defects, micro-scans (CT), immunohistochemical studies for phenotypic bone tissue markers, and histological evaluations showed that the defects were partially reconstructed in all test groups, while the group whose defects were filled with alginate–chitosan scaffold composites together with BMP-2 had the highest defect reconstruction of about 71.56 ± 19.74%. In another study conducted by Soumya et al. [128], lyophilized alginate-o-carboxymethyl chitosan scaffolds loaded with an extract of Cissus quadrangularis (Veld Grape) were prepared. The composite scaffolds were seeded with MSCs and their ability to induce osteogenic differentiation of MSCs and formation of mineral-deposited ECM was investigated. As reported by Soumya et al. [128], the composite scaffolds loaded with herbal extract exhibited the highest cell proliferation and attachment. In addition, the composite scaffolds loaded with herbal extract induced the differentiation of MSCs into osteoblasts and promoted the formation of mineralized ECM after 14 days of incubation.

**Figure 6:**
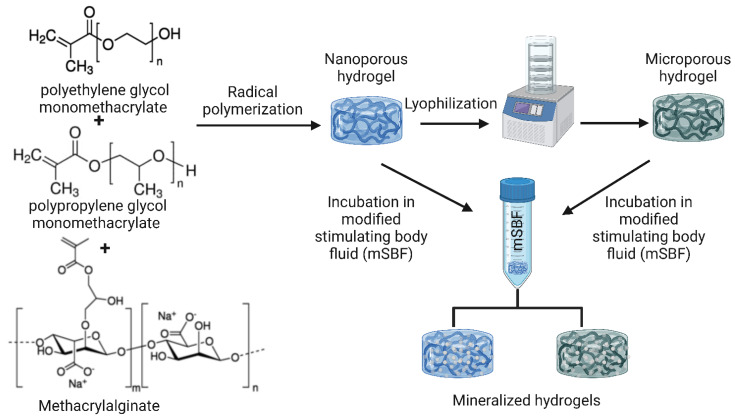
PPGmM and PEGmM were chemically crosslinked to form hydrogels using MA as a crosslinking agent. The resulting hydrogels (nanoporous hydrogels) and hydrogels freeze-dried to microscopic pores (microporous hydrogels) were incubated in mSBF to induce biomineralization [129]. The image was created using Biorender.

Composites of alginate and synthetic polymers have also been reportedly prepared. Nanocomposite hydrogels with micro- (lyophilized) and nanoscale (non-lyophilized) pores were prepared by incorporating alginate together with polyethylene glycol monomethacrylate (PEGmM) and polypropylene glycol monomethacrylate (PPGmM) crosslinked via methacrylalginate (MA), as shown in Figure 6. After one week of incubation in modified stimulating body fluid (mSBF), the composite caused mineralization by the formation of apatite crystals. In addition, it was found that increasing the proportion of the synthetic polymer compared to alginate enhanced mineralization due to the subsequent increase in hydrophobic nature, pore size, and charge density [129].

**Figure 7:**
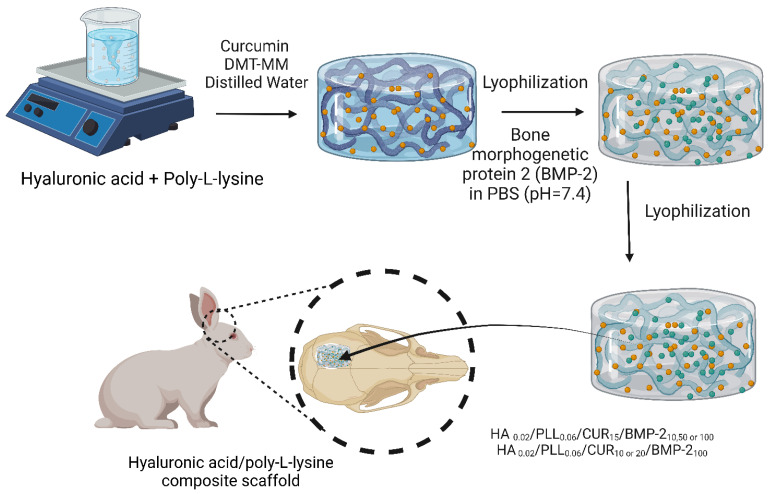
A flow chart showing the preparatory steps for manufacturing HPC-2, HPB-3, and HPCB-1~5 and in vivo investigations carried out on an 8 mm induced defect of rabbit calvaria [130]. The image was created using Biorender.

Hyaluronic acid has been reportedly crosslinked to hydrogel-based composites loaded with bioactive molecules for BTE applications (Figure 7). A hyaluronic acid/poly-L-lysine composite scaffold loaded with curcumin and BMP-2 was prepared. In vitro studies on MG63 osteosarcoma cells revealed that the composite scaffold allowed the sustained release of curcumin and BMP-2 over a 28-day period and marked proliferation and osteogenic differentiation of MG63 cells. In vivo evaluations of defects in rabbit skulls confirmed the remarkable synergistic bone healing effects between curcumin and BMP-2 [130].

### 5.2. Immunomodulatory Roles of Polymer Scaffolds Utilized in Bone Regeneration Applications

Implantation of scaffolds made of natural or synthetic polymers often results in the development of an inflammatory response. As a result of this inflammatory response, various biochemical signals have been triggered that cause the recruitment of different types of immune cells at the implantation site. After recruitment at the implantation site, monocytes differentiate into macrophages under the influence of cytokines synthesized and secreted by other immune cells. The differentiated macrophages then begin to attach to the surface of the implant, and the plasticity of the attached macrophages is largely controlled by the physicochemical properties of the implanted scaffold, which has a significant effect on the bone regeneration process [55]. It has been reported that the triggered immune response to the implanted biomaterial-based scaffolds is mainly due to the interactions between various proteins and the implanted scaffold. The surface properties of the implanted scaffold may alter the amount, type, and manner of protein adsorption and even lead to changes in protein conformation [131]. All of the aforementioned parameters may ultimately lead to the regulation of immune cell activities.

Macrophage-mediated regulation of bone formation in healthy and weakened immune systems has been demonstrated [132]. Macrophages exert their immunomodulatory effects via secreted cytokines and extracellular vesicles (EVs). EVs contain microRNAs (miRNAs) and have been shown to be central to the osteoblastic differentiation of MSCs [133]. It has been suggested that the functionality of EVs could be altered by genetic modification of parental cells to induce osteoinduction and bone regeneration [134]. EVs from M1 macrophages have a different miRNA cargo compared to M2 macrophages. M2 EVs can promote bone repair/regeneration, whereas M1 EVs can inhibit bone repair by negatively regulating the BMP pathway [135]. Cytokines secreted via the phenotypes of M1 and M2 macrophages have a great influence on osteogenesis. M1 macrophages produce several proinflammatory cytokines, including IL-6, IL-1β, TNF-α, and INF-γ [136,137]. INF-γ is known to inhibit collagen synthesis by osteoblasts [138]. TNF-α and IL-1β are responsible for inhibiting the production of alkaline phosphatase (ALP), which negatively affects ECM synthesis and mineralization [139]. On the other hand, M2 macrophages are responsible for the synthesis of anti-inflammatory cytokines, including IL-1RA, IL-10, and TGF-β [136,137]. IL-10 has been shown to stimulate osteogenic differentiation [140]. TGF-β has been shown to upregulate signaling through bone morphogenetic protein (BMP), which is required for osteoblast differentiation [136].

The immunomodulatory properties of MSC in bone repair are well documented. The paracrine effects of MSCs in immunomodulation are due in part to their secreted EVs. When MSCs migrate into the scaffold bed, they are exposed to a variety of inflammatory signals that influence the immunomodulatory function of MSC EV in tissue repair [141]. It is known that the immunomodulatory effect of MSCs is exerted by the secretion of regulatory cytokines or by direct cell–cell contact [142]. During the bone regeneration process, the secretion of cytokines by MSCs can vary significantly depending on the healing phase, resulting in the regulation of proliferation, activation, and migration of other immune cells [143]. Based on the levels of anti-inflammatory and proinflammatory cytokines found in the microenvironment in which they reside, MSCs synthesize and secrete cytokines, including TGF-β, which causes stimulation of regulatory T cells (Tregs) [144]. In addition, MSCs have been shown to secrete anti-inflammatory TNF-stimulated gene protein 6 (TSG-6), which prevents neutrophil migration by inhibiting the binding of C-X-C motif chemokine ligand 8 (CXCL8) with heparin [145].

#### 5.2.1. Factors Affecting Polymer Scaffolds-Based Immunomodulation for Bone Regeneration

Most previous studies performed in this manner focused on the control of osteoblast differentiation without considering the provoked immune response. However, more recent studies have focused on controlling the bone regeneration process by focusing on the roles of MSCs, neutrophils, and macrophages via manipulating the chemical and physical properties of implanted scaffolds or loading the scaffolds with cytokines or biomolecules [146]. This promotes osteogenesis through immune modulation. When developing polymer scaffolds, some factors must be considered to successfully promote osteogenesis while alleviating the accompanied inflammatory response, such as:

(1) Scaffold stiffness: the control of scaffold stiffness is critical in the development of polymeric bone regeneration scaffolds because it affects proliferation [147], migration [148], differentiation [147], contractility [149], and the fate of osteoprogenitor cells during the bone regeneration process. In addition, the rigidity of the scaffold may also influence the inflammatory response of the host to the implant. Since macrophage polarity is directly linked to their function, scaffold stiffness can control their phenotype by regulating their cytokine secretion, spread, and cytoskeleton, causing macrophages to promote either inflammation or tissue regeneration [150]. In a study by Friedemann et al. [151], the effect of scaffold stiffness on human macrophages was investigated using 3D collagen/glycosaminoglycans (GAGs) scaffolds with different stiffnesses. It was found that macrophages differentiated into a non-inflammatory M2 phenotype by expressing fewer inflammatory cytokines such as IL-12 and TNF-α and more anti-inflammatory cytokines such as IL-10, suggesting that macrophages can sense the stiffness of implanted polymer scaffolds.

(2) Surface roughness: it has been shown that differentiation of macrophages into the M2 phenotype is favored on rough surfaces and that neutrophils are more likely to attach to rough surfaces than to smoother surfaces, suggesting that differentiation of both macrophages and neutrophils is influenced by the nano- and microstructures on the scaffold surface [152,153]. In a study conducted by Chen et al. [154], the effect of nano- and micro-scaffolds attached to various polymers commonly used in tissue engineering applications was tested on human macrophages. It was found that parallel imprinted gratings with a diameter of 250 nm to 2 μm triggered the expression of anti-inflammatory cytokines.

(3) Porosity and pore size: osteogenesis was proved to be highly dependent on scaffold pore size and porosity [155], these results were observed both in vivo and in vitro as scaffolds with an average pore size of 200 to 350 μm and a porosity of 80 to 88% was considered ideal for bone formation [156]. It has been demonstrated that increasing the pore size of the polymer scaffold led to a decrease in the provoked immune response and thus to better healing of bone tissue. In a study conducted by Garg et al. [157], it was demonstrated that pore size plays an important role in the polarization of bone marrow-derived macrophages when tested for polydioxanone nanofibers. The expression of markers of the anti-inflammatory M2 phenotype increased and the expression of markers of the inflammatory M1 phenotype decreased when the mean pore size of the electrospun nanofibers was increased. Furthermore, porosity and pore size control oxygen delivery within the implanted polymer scaffold, and low oxygen delivery promotes inflammation at the implantation site [158].

(4) Surface charges: controlling the surface charges of polymer scaffolds is critical because surface charges have been found to influence protein adhesion, which ultimately affects the host’s immune response to the implanted scaffold [159]. Scaffold surfaces with hydrophilic anionic or neutral nature, when exposed to macrophages, were found to stimulate the production of IL-8, IL-6, IL-1β, and TNF-α, leading to classical macrophage activation, whereas hydrophilic cationic scaffold surfaces lead to alternative macrophage activation [160].

#### 5.2.2. Approaches for Enhancing Immunomodulatory Effects of Polymer Scaffolds Utilized in Bone Regeneration Applications

There are several approaches to enhance the immunomodulatory effects of polymer scaffolds used in bone regeneration, such as:

(1) Incorporation of ECM-derived biomaterials: the use of biomaterials derived from native ECM has been shown to activate the polarization of macrophages toward the M2 phenotype, resulting in enhanced bone remodeling [161]. Various ECM-derived biomaterials can be incorporated into polymeric scaffolds to enhance their immunomodulatory effects, such as a demineralized bone matrix [162], collagen [161], and fibrinogen [163]. In a study conducted by Taraballi et al. [161], the immunomodulatory effect of collagen scaffolds functionalized with chondroitin sulfate was investigated both in vitro in bone marrow-derived macrophages and in vivo in the adult Lewis rat model. According to the results of Traballi’s in vitro studies, macrophages cultured on the scaffolds showed upregulation of anti-inflammatory M2 genes and downregulation of proinflammatory genes. The results of the in vivo studies showed significant downregulation in proinflammatory genes 3 days after implantation.

(2) Incorporation of bioactive metal ions: it has been reported that bioactive metal ions can alleviate undesirable inflammatory reactions after the implantation of polymer scaffolds. Such metal ions include Mg^2+^, Ca^2+^, and Sr^2+^ [164]. It has been reported that these bioactive metal ions can modulate the inflammatory microenvironment of the scaffold. This can be attributed to the released metal ions in vivo, which can interact with the immune system through Toll-like receptors (TLRs) [165]. This regulates the induced inflammatory response and cellular activation. To investigate the immunomodulatory effect of Mg^2+^ ions, Cifuentes et al. [166] prepared a poly-D-L-lactic acid (PDLLA) matrix loaded with Mg^2+^ and tested the behavior of macrophages, osteoblasts, and MSCs seeded on them. The Mg^2+^-loaded PDLLA matrix was reported to downregulate ALP activity, VEGF and fibronectin synthesis, and expression of inflammatory chemokines such as macrophage inflammatory protein-1 (MCP-1) compared with the PDLLA matrix, which resulted in increased expression of this inflammatory chemokine.

(3) Incorporation of bioactive molecules: to reduce the intensity of the inflammatory response associated with scaffold implantation, some bioactive molecules can be introduced into the scaffolds, such as IL-4 [167], IL-10 [168], CD200 [169], and some anti-inflammatory drugs such as dexamethasone [170], indomethacin [171], and Resolvin D1 [172]. In a study by Zhang et al. [162], gellan gum (GG) bead scaffolds were prepared and loaded with different concentrations of IL-4 (100, 200, and 300 ng/mL). In vitro studies using transwell cocultures performed with bone mesenchymal stem cells (BMSCs) and human macrophage RAW 264.7 cell lines showed that the expression of TGF-β1R was significantly higher in GG-IL-4 groups compared with other groups. In addition, staining with Alka-line phosphatase (ALP) and Alizarin Red S (ARS) revealed that GG-IL-4 groups had significantly higher activity of ALP and a higher level of calcium deposition compared with other groups. Immunohistochemical studies performed in vivo on male Sprague Dawley rats showed decreased expression of the proinflammatory cytokine M1 TNF-α. In addition, micro-scans (CT) revealed that rats treated with GG-IL-4 scaffolds had the highest percentage of defect filling among all experimental groups. In conclusion, loading with IL-4 significantly improved the bone regeneration capacity of scaffolds by polarizing macrophages toward the M2 anti-inflammatory phenotype.

## 6. Novel Designs of Polymer Scaffolds for Bone Tissue Engineering

**Figure 8:**
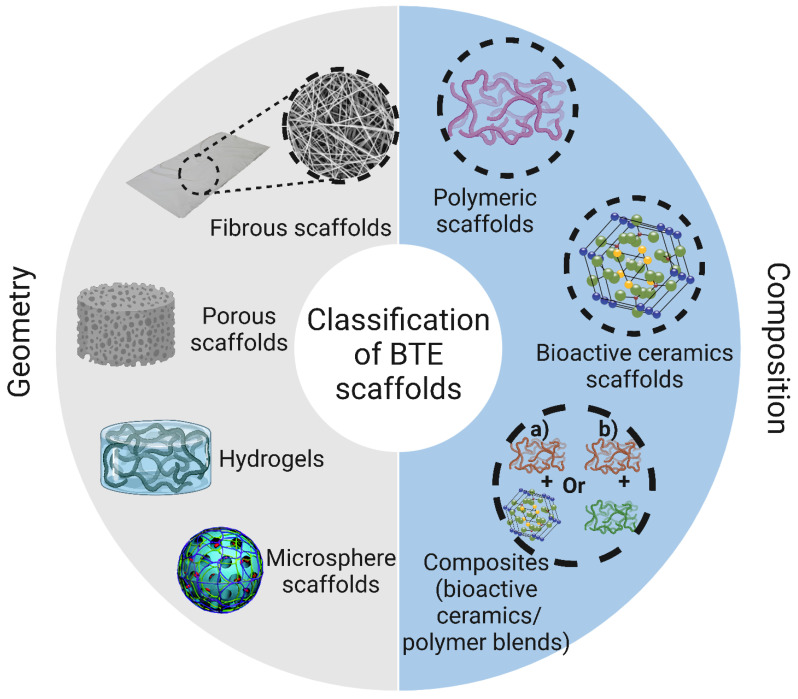
Classification of polymeric scaffolds used in bone tissue engineering according to their geometry and composition. The image was created using Biorender.

Scaffolds used for BTE applications can be classified based on their geometry into (i) porous scaffolds (also known as sponge or foam scaffolds) [173], (ii) hydrogels [174], (iii) fibrous scaffolds [175], and (iv) microsphere-based scaffolds [176]. Furthermore, based on their composition, BTE scaffolds can be divided into (i) polymeric scaffolds [177], (ii) ceramic-based bioactive scaffolds [178], and (iii) composites [179]. The classification of BTE scaffolds is shown in Figure 8.

### 6.1. Porous (Sponge or Foam) Scaffolds

Three-dimensional porous polymer-based scaffolds are characterized by a high degree of porous interconnectivity and are extremely beneficial in the TE field. These scaffolds can support ECM formation, leading to enhanced biomimicry. Porous scaffolds exhibit several benefits related to cell proliferation, including (i) enhanced nutrient, waste, and oxygen diffusion through their interconnected pores; (ii) they assist cells in synthesizing their ECM; (iii) they only allow cell proliferation as a monolayer; (iv) their defined pore dimensions limit the formation of cell clusters to a certain size, thus preventing tumor development [173]. Various methods have reportedly been used to fabricate sponge scaffolds with macroscopic interconnected pores, including freeze-drying, salt leaching, laser sintering, and rapid prototyping (RP) techniques [180]. Reportedly, a combination of freeze-drying and particle leaching has been used to fabricate sponge scaffolds. In this hybrid technique, the pore size can be adjusted by varying the temperature, viscosity of the polymer solution, and salt concentration [181]. Both pore size and morphology appear to be critical factors affecting the proliferation and osteogenic differentiation of cells on the surface and within the pores of sponge scaffolds. Although pores are 10–50 µm in size [182], osteoblasts have been found to prefer a pore size of 100–200 µm to synthesize mineralized bone tissue that allows phagocytic clearance of harmful bacteria at the fracture site and promotes cellular colonization and neovascularization [182]. In contrast, pore sizes less than 100 µm have been associated with the formation of non-mineralized tissue [183]. Regarding pore morphology, Van Bael et al. [184] found that scaffolds with hexagonal pores promoted the most cellular proliferation, followed by scaffolds with rectangular pores, and the least cellular proliferation was shown by scaffolds with triangular pores. These differences can be attributed to the higher number of corners and the small distance between arcs in hexagonal pores compared to other morphologies. However, the authors reported that ALP activity was highest in scaffolds with triangular pores.

The scaffold’s porosity affects some parameters of the framework, including (1) mechanical strength: it has been shown that an increase in the porosity of the framework leads to an exponential decrease in the mechanical strength of the framework [185]. In addition, it has been reported that the molecular weight of the polymer contributes greatly to the porosity and pore size of the scaffold, thus affecting the mechanical strength [186]. Other parameters such as pore morphology, pore size distribution, and pore homogeneity also affect the mechanical strength of the scaffold [187]. In a study conducted by Serra et al. [188], it was reported that PLA/PEG/Cap glass composite scaffolds with orthogonal pore structures have higher compressive strength than PLA/PEG/Cap glass composite scaffolds with displaced double-layer patterns. (2) Scaffold degradation rate: to investigate the effect of pore geometry and porosity on the degradation rate of polymer scaffolds, Khajehmohammadi et al. [189] prepared gelatin-coated 3D-printed PCL scaffolds with different porosities (40, 50, and 60%) and different pore geometries (square, star, and gyroid). When comparing the different porosities, it was found that scaffolds with star-shaped geometry showed the highest weight loss compared to gyroid and square pore geometries. In addition, scaffolds with a porosity of 40% were found to have significantly higher weight loss compared to scaffolds with porosities of 50% and 60%.

### 6.2. Hydrogel-Based Polymer Scaffolds

Hydrogels are polymer-based scaffolds consisting of a three-dimensional network of polymer chains that are either covalently or non-covalently linked. Hydrogels are of great advantage in TE because they provide a microenvironment that can support cell proliferation and subsequently rapid tissue formation. In addition, hydrogels can serve as encapsulating agents for various drugs, cells, and biomolecules to achieve sustained release of the desired drug or biomolecules, or even to transport cells to the defective site [174,190]. Hydrogels have been shown to have good bioabsorption capacity and to fuse with the surrounding host tissue to a high degree, thus not requiring additional surgical procedures to remove the implant and not causing inflammation [191]. Both natural and synthetic polymers can be processed into hydrogels. Hydrogels based on natural polymers are very advantageous when used in TE because of their close resemblance to the ECM, biodegradability, non-cytotoxicity, non-immunogenicity, and ability to promote cell adhesion and proliferation and induce new tissue formation. Hydrogels can be prepared by chemical, physical, or radical crosslinking of polymers [192]. Lindsey et al. [193] prepared an injected collagen-based hydrogel for the reconstruction of dorsal nasal bone defects in rats. Six weeks after implantation, a superficial layer of thin bone tissue formed around the defect site, whereas only less than 7% of the healing area was observed in the same control. Several synthetic biodegradable polymers have been processed into hydrogels, including polyvinyl alcohol (PVA), poly(lactic acid) and its copolymers, polyacrylamide (PAM), and polyethylene glycol (PEG) [194]. Compared to natural polymers, the porosity, mechanical properties, and degradation rate of synthetic polymers can be better controlled, allowing them to be tailored for specific applications. Hydrogels based on synthetic polymers serve as promising carriers for bioactive molecules and drugs when used in BTEs. Lee et al. [195] constructed a novel hydrogel of poly aldehyde guluronate (PAG) and adipic acid dihydrazide that served as a carrier for primary rat cranial osteoblasts to reconstruct spinal defects in rats. Nine weeks after surgery, noticeable bony mineral-deposited tissue formed.

### 6.3. Fibrous-Based Polymer Scaffolds

Nanofiber scaffolds have recently attracted much attention in the field of TE due to their high biomimicry. Three main techniques are used to process polymer into nanofibers: electrospinning, phase separation, and self-assembly. The most commonly used technique is electrospinning [196]. The potential of nanofibers produced by self-assembly and phase separation has not been thoroughly investigated in comparison with nanofibers produced by electrospinning in TE. The reasons why nanofibers are promising candidates for TE are their impressive surface-to-volume ratios and their interconnected pores that support cell attachment, infiltration, differentiation, and proliferation [197]. For these reasons, nanofibers have been used in various applications, such as bone, cartilage, and ligament reconstruction, wound healing, neuronal TE, and as a carrier material for the sustained release of various biomolecules and drugs [198,199,200,201]. Reportedly, a variety of natural and synthetic polymers have been processed into nanofibers. Natural polymers are used in BTE applications because they are highly biocompatible and exhibit a high degree of biomimicry [202,203]. The mechanical properties of natural polymers can be improved by crosslinking [204]. For example, collagen-based nanofiber scaffolds can be crosslinked during or before electrospinning in various ways, such as ultraviolet (UV) irradiation or stabilization with epoxy compounds, methanal, and glutaraldehyde vapors [205,206,207]. Zhou et al. [208] fabricated a collagen-based electrospun nanofiber scaffold crosslinked with glutaraldehyde vapor. The resulting nanofibers exhibited a mechanical strength of 6.72 ± 0.44 MPa, which enabled their use as skin graft substitutes. Several synthetic polymers have reportedly been processed into nanofibers, including PCL, PLA, PGA, PEO, and PVA. The main advantages of electrospun fibers from synthetic polymers over fibers from natural polymers are their better mechanical properties, spinnability, and the fact that they are cheaper and more readily available [209]. Due to its exceptional biodegradability, biocompatibility, and mechanical properties, PCL is widely used in applications where mechanical stiffness is a must. PCL-based nanofiber scaffolds were fabricated by Gomes et al. [210]. These nanofibers have a diameter of 1833 ± 369 nm, extensibility of 587 ± 162%, and elasticity of 6.7 ± 0.4 MPa, making them ideal for TE.

### 6.4. Microsphere-Based Polymer Scaffolds

Due to their growing reputation as good carriers for biomolecules and drugs, 3D microsphere-based scaffolds are widely used for TE applications to deliver drugs and biomolecules to defective tissues and promote cell growth [83,211]. The most widely used method for fabricating microsphere-based scaffolds is sintering, which results in so-called sintered microsphere scaffolds (SMSs). The sintering technique can be either heat-induced or solvent-based. In heat-induced sintering, the polymeric microspheres prepared by single/double emulsification are placed in a Teflon reactor line, which is then heated to a temperature above the glass transition temperature (Tg) of the polymer for several hours, and then the mold is removed. The solvent-based approach uses a solvent-induced fusion of microspheres to obtain 3D scaffolds. Acetone and methylene chloride are used as solvents in this process. The main advantage of solvent-based sintering over heat-induced is that the solvent-based approach is more suitable for fabricating scaffolds loaded with heat-sensitive biomolecules [212]. In order to design SMSs suitable for BTE applications, a number of crucial issues need to be considered, such as the Tg of the polymer, crystallinity, surface tension, and molecular weight, as well as heating temperature (in the case of heat-induced sintering) and solvent concentration (in the case of solvent-based sintering). For example, higher heating temperatures and times result in microspheres with lower porosity and average pore size, which may limit cell migration and neovascularization [94]. The most commonly used polymer for the fabrication of SMS is PLGA because it is exceptionally biodegradable and biocompatible and has tremendous drug-loading capacity. Other polymers used for the preparation of SMS include polycaprolactone (PCL), polyphosphazenes, and chitosan due to their structural similarity to PLGA [212,213,214,215]. Kofron et al. [216] developed PLGA-SMS using semi-crystalline and amorphous PLGA for the reconstruction of induced bone defects in rabbits. Both scaffolds showed high similarity to the natural bone in terms of porosity and average pore size. However, the authors reported that after six months of implantation, scaffolds made of amorphous PLGA promoted greater bone tissue formation compared to scaffolds made of semi-crystalline PLGA, suggesting that amorphous PLGA is more suitable for BTE applications.

### 6.5. Bioactive-Composite-Based Scaffolds

Composites are resulted from combining two or more different materials. Composites can be (1) polymer–polymer composites, (2) polymer–ceramic composites, or (3) polymer–bioactive material composites. Poly(lactic-co-glycolic acid) (PLGA) is a copolymer of lactic acid and glycolic acid and is commonly utilized in BTE applications [179]. However, the acidic byproducts resulting from its degradation have a toxic effect on the surrounding native tissue when implanted in vivo. PLGA–polyphosphazene composites were developed which gave almost neutral degradation byproducts. Polymer–ceramic composites are frequently used in BTE applications due to their high resemblance to natural bony tissue. Collagen–HAp composites showed a superior bone regenerating effect when compared to collagen and HAp alone. Additionally, the mechanical properties of the scaffolds were greatly enhanced upon the incorporation of bioactive ceramics while reducing their elasticity [217].

## 7. Bioscaffold Fabrication Techniques

**Figure 9:**
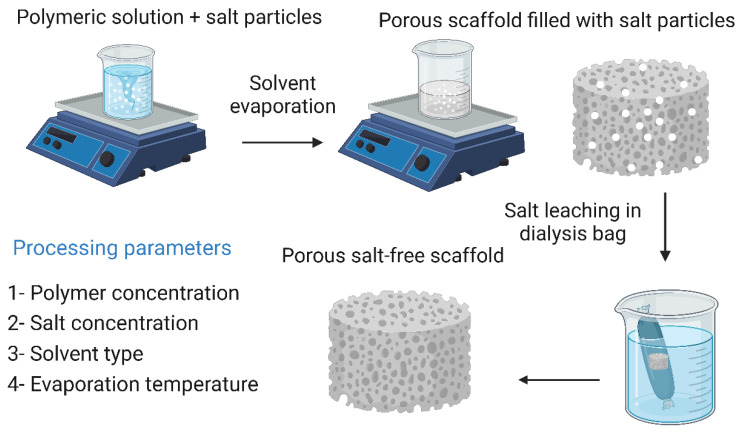
Solvent casting and particulate leaching technique and its processing parameters. The image was created using Biorender.

Three-dimensional scaffold fabrication technologies are classified into conventional and rapid prototyping (RP) techniques [13]. Conventional techniques, also known as subtractive manufacturing, rely on removing parts of the main structure until the desired shape is attained. However, some drawbacks are linked to conventional techniques including not being able to fully control the shape and dimensions of the resulting scaffolds, lacking the ability to design tailored internal structures such as vessels [218], and the cytotoxic effects of the organic solvents utilized in these processes [13]. Conventional scaffold fabrication techniques and the parameters that affect the resulting scaffolds are listed in Figure 9. RP techniques have emerged recently as promising approaches to compensate for the drawbacks linked to conventional means. RP technologies are known as additive manufacturing as they rely on the layer-by-layer addition of fabricating material to obtain the desired 3D scaffold [219]. RP techniques are advantageous over conventional techniques because the shape, dimensions, and mechanical properties of the resulting 3D scaffold can be fully controlled, enabling the creation of 3D scaffolds with a high degree of biomimicry. In addition, RP technologies allow the use of two or more materials on the surface, interface, or entire scaffold [19]. Operating RP techniques is done via computer-aided design/computer-aided manufacturing setups (CAD/CAM), consisting of three parts: a scanning device that converts the architecture of the scaffold into digital data that can be processed by a computer, integrated software that converts these digital data into commands for the fabrication machinery, and finally fabrication machinery that converts these sets of commands into the desired 3D scaffold [220].

### 7.1. Solvent Casting and Particulate Leaching

Solvent casting and particulate leaching yields highly porous frameworks with controllable pore sizes. Briefly, a polymeric material is dissolved in a suitable solvent along with salt particles of a known size, then the solvent is evaporated, leaving the salt particles in the polymer matrix. The salt particles are then leached out by immersing the polymer matrix in distilled water, resulting in a highly porous scaffold whose pores have the same dimensions as the salt particles. This technique has the advantage of being easy to perform and does not require sophisticated equipment. In addition, the malleability of the pore size makes it easy to produce structures that resemble natural bone tissue, which increases the biomimicry of the obtained scaffold [13,218]. Cao et al. [98] reported that they fabricated three-dimensional PGA/β-TCP composite scaffolds by solvent casting and particle leaching techniques. The fabricated scaffolds exhibited high porosity and interconnectivity of pores. In in vivo tests to evaluate their ability to reconstruct severe bone damage to the medial epicondyles of the femur of rats, imaging, and histological studies showed that new bone tissue began to form 14 days after implantation and bone formation was complete after 30 days. By day 90, the bone replacement was complete. However, this technique has several disadvantages, including the inability to construct more complex geometries, and the remaining solvent may have cytotoxic effects on cells, reducing the overall biocompatibility of the scaffold [98,221]. The solvent casting and particulate leaching technique along with its processing parameters are illustrated in Figure 9.

### 7.2. Freeze-Drying

**Figure 10:**
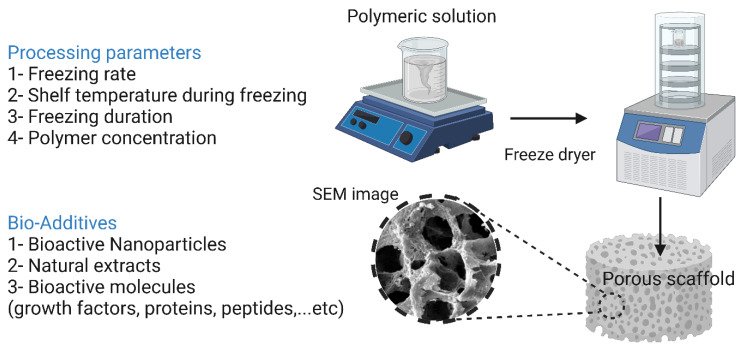
Freeze-drying technique and its processing parameters and additives. The image was created using Biorender.

Freeze-drying involves freezing a polymeric solution via liquid nitrogen until the solvent completely evaporates. This technique is very advantageous when heat-sensitive biomolecules are incorporated into the scaffold, as high temperatures can lead to a reduction or total loss of their biological activity. In addition, this technique makes it relatively easy to control the pore size by manipulating the freezing conditions [19]. Xu et al. [222] reported the preparation of a bioglass–collagen–phosphatidylserine composite scaffold by freeze-drying. As reported by the authors, the scaffold had an average pore size of 300 μm with relatively high interconnectivity. Phosphatidylserine is able to form stable complexes with both calcium and phosphate, leading to the formation of HAp nuclei, a property unique to most phospholipids. This ability to form HAp nuclei is a major contributor to its use in bone regeneration. In in vitro tests with rat MSCs, after 21 days of cultivation on the scaffold, the cells began to proliferate and showed osteogenic behavior. In vivo evaluations on rats’ femurs showed that the scaffold was both biocompatible and osteoconductive after six weeks of implantation. However, the use of the freeze-drying technique in BTE has some shortcomings, such as the use of an organic solvent that may have cytotoxic effects on cells, the high energy requirement, the extremely time-consuming procedure, and, most importantly, the formation of miniature pores (in the range of 15 to 35 μm) with irregular morphology [179]. Changing the freezing conditions could provide a solution to the last problem, since varying the freezing temperature (−10 °C to −70 °C) and introducing an annealing step greatly increases the ice crystal growth rate. As reported by Murphy et al. [70], the application of these modifications resulted in an increase in the pore size of collagen–glycosaminoglycan scaffolds to 85–325 μm. The freeze-drying technique along with its processing parameters and additives are illustrated in Figure 10.

### 7.3. Thermally Induced Phase Separation Methods 

**Figure 11:**
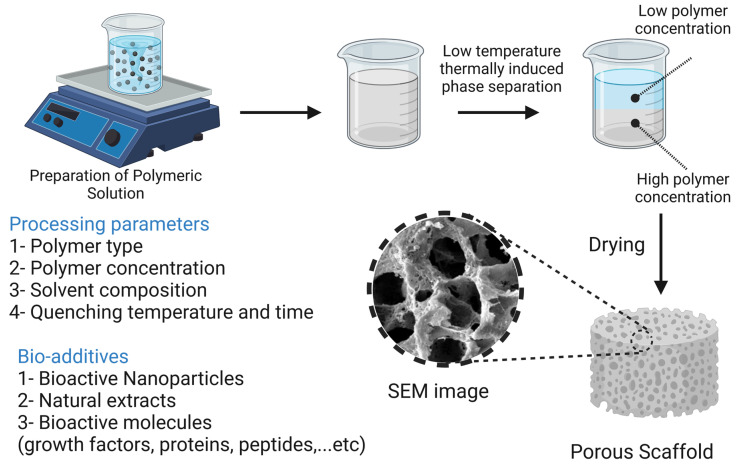
The thermally induced phase separation technique and its processing parameters and additives. The image was created using Biorender.

At extremely low temperatures, a polymeric solution is quenched and segregated into two separate phases, where the phase with the higher polymer content is solidified while the phase with the lower polymer content is precipitated, creating a highly porous nanofiber structure. The superiority of the TIPS technique is evident in the fabrication of scaffolds loaded with thermolabile biomolecules [19]. Smith et al. [223] reported the fabrication of PLLA scaffolds loaded with nanoparticles loaded with recombinant human BMP-7. PLLA scaffolds were fabricated with fibers ranging from 50 to 500 nm in diameter using the TIPS technique. The porogen leaching approach was then used to load the scaffolds with NPs that acted as carriers for recombinant human BMP-7. In in vitro assays, the scaffold-assisted release of BMP-7 resulted in enhanced osteogenic differentiation of cells. Qui et al. [224] reported the preparation of PLLA/PCL-silica-NP composite scaffolds that serve as a delivery system for dexamethasone. PLLA/PCL nanofibers were prepared using the TIPS technique, then prepared aminated mesoporous SiO2 NP (serving as a carrier for dexamethasone) was deposited on the scaffolds by electrophoresis. In in vitro assays using bone marrow-derived MSCs, measurements of ALP activity, ECM mineralization, and osteocalcin gene expression revealed that the composite scaffolds greatly enhanced the cells’ ability to undergo osteogenic differentiation. In vivo testing on Sprague Dawley rat calvaria defects showed significant promotion of calvaria defect healing. The TIPS technique along with its processing parameters and additives are illustrated in Figure 11.

### 7.4. Gas Foaming Methods 

**Figure 12:**
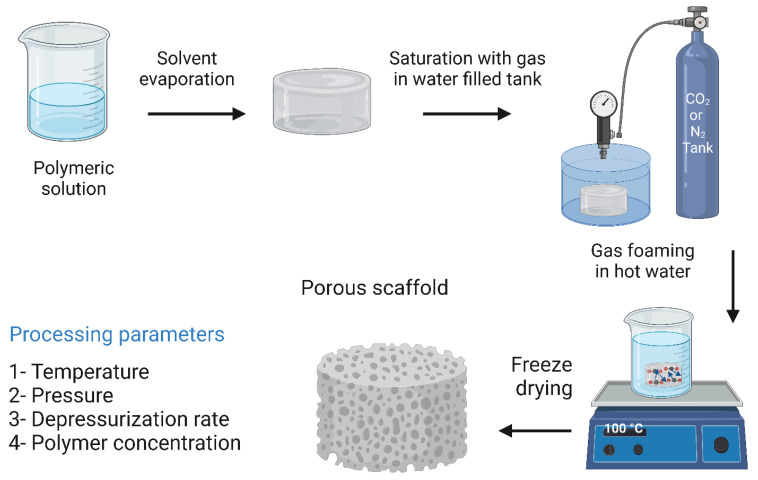
The gas foaming technique and its processing parameters. The image was created using Biorender.

Gas foaming technology does not utilize toxic organic solvents. Instead, inert gases such as carbon dioxide and nitrogen are used. The process begins with compressing biodegradable polymers immersed in water using an inert gas (nitrogen) to a point where the polymer is completely saturated with gas bubbles, resulting in sponge-like structures with an average pore size of 30 to 700 μm and a porosity of about 85% [13]. Giannitelli et al. [16] reported this technique for the fabrication of a functionally graded material (FGM) composed mainly of polyurethane, which was used for the reconstruction of oral and maxillary bone tissue damage. The fabricated scaffold consisted of two main regions, a high-density outer layer that serves as a barrier to gingival tissue growth, and a less dense inner core with interconnected pores that allow osteogenesis. In vitro tests with bone marrow-derived MSCs have shown that the scaffolds can support cell viability and attachment. Although the mechanical properties of the scaffolds do not match the mechanical properties of natural spongy bone, they can still withstand the stresses applied at the implant site. The gas foaming technique has some disadvantages, such as the high temperatures applied when compressing the mold and the lack of porosity at the dense surface of the scaffold. To increase the porosity of the scaffolds, Harris et al. [225] fabricated a PGLA scaffold by combining gas foaming and particle leaching, which resulted in scaffolds with increased porosity of up to 97%. The gas foaming technique along with its processing parameters are illustrated in Figure 12.

### 7.5. Sol–Gel Methods 

**Figure 13:**
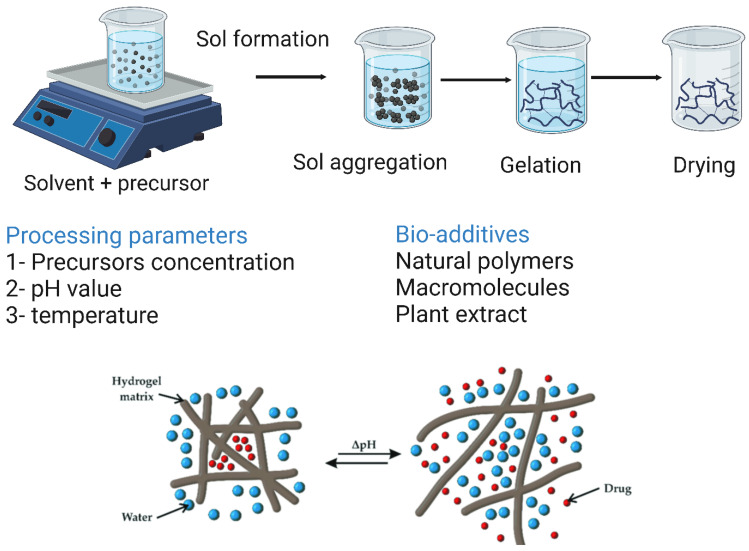
The sol–gel formation technique and its processing parameters and additives. The image was created using Biorender.

Sol–gel formation is a technique in which metal alkoxides are polymerized in an inorganic manner. Briefly, the formation of a solution (sol) is done by adding a surfactant, then the sol is condensed and finally, the condensed product is gelled (gel). Chen et al. [226] reported the preparation of sodium oxide-containing BG ceramics by a hybrid sol–gel process, which exhibits enhanced mechanical stiffness without compromising its biodegradability. The sol–gel formation technique along with its processing parameters and additives are illustrated in Figure 13.

### 7.6. Electrospinning Methods 

Electrospinning is a technique in which a polymeric solution loaded into a syringe is drawn off under the action of high voltages to form nanofibers. These nanofibers are collected on the surface of a collector, and the resulting nanofibers can support cell growth and attachment through protein binding sites capable of binding to cell surface receptors. A typical electrospinning system consists of four main parts: a spinneret with a metal needle, a syringe pump, a high-voltage power supply, and a collector. The fibers are formed as follows: once the applied electric field exceeds the surface tension of the polymer droplet at the tip of the needle, the liquid is continuously ejected under the effect of electrostatic repulsion and collected at the surface of a collector with the opposite charge, forming nonwoven fibers. During the electrospinning process, the solvent is evaporated [218,227].

**Figure 14:**
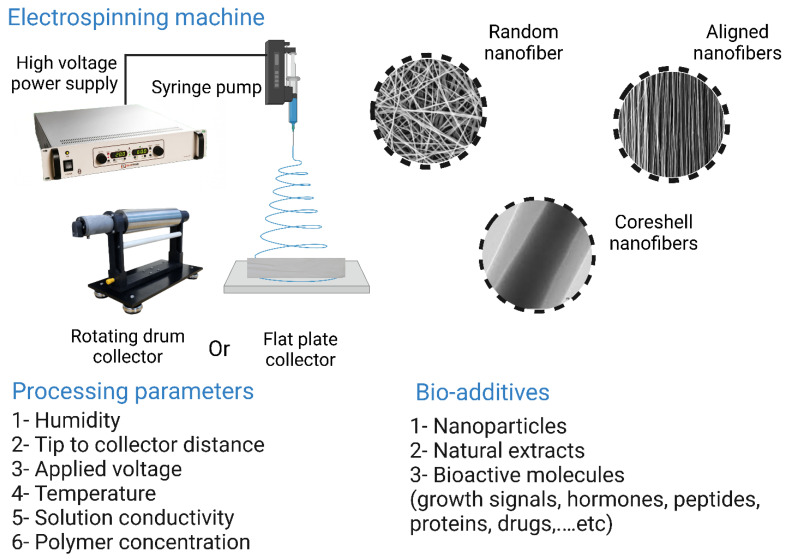
The electrospinning technique and its processing parameters, additives, and nanofiber types. The image was created using Biorender.

Electrospinning can be used to process various materials to obtain scaffolds with desired architecture and porosity, e.g., fibrous scaffolds with fiber diameters in the micro or nano range [19]. Due to the inferior mechanical properties of pure chitosan scaffolds and the poor cell adhesion of PCL fibers, Yang et al. [228] fabricated biocomposite scaffolds from a combination of chitosan and PCL using electrospinning, and reported that they exhibited suitable mechanical properties and supported the proliferation and attachment of MC 3R3-E1 cells in in vitro assays. In vitro results also indicated that the biocomposite scaffolds showed higher ALP activity, ECM mineralization, and increased expression of OP. Another important feature of electrospinning is the functionalization of nanofiber scaffolds by introducing bioactive molecules. Li et al. [229] developed an electrospun nanofiber scaffold loaded with NPs that served as a dual vehicle for dexamethasone and BMP-2. The activity of BMP-2 was maintained by encapsulating it in bovine serum albumin (BSA) NPs. In in vitro assays, the fabricated nanofiber scaffolds were able to promote the osteogenic differentiation of cells. In vivo studies in rats revealed that the nanofiber scaffolds were able to significantly heal defects on rat calvaria. However, the use of organic cytotoxic solvents remains one of the most common drawbacks of electrospinning [19]. The electrospinning technique along with its processing parameters, additives, and nanofiber types are illustrated in Figure 14.

### 7.7. Stereolithographic Methods 

**Figure 15:**
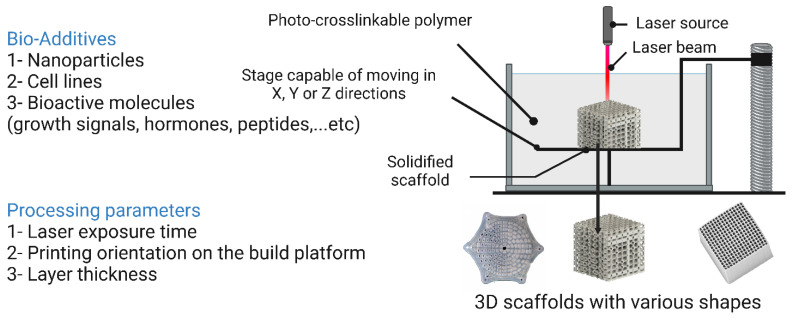
Stereolithography technique and its processing parameters and additives. The image was created using Biorender.

Stereolithography (SL) was first introduced by Charles Hull in 1986 and involves the layer-by-layer deposition of ultraviolet (UV)-curable materials to obtain solidified structures [230]. The SL technique provided a solution to the drawbacks associated with conventional approaches, such as wasting large amounts of raw materials by disposing of unused parts and scratches caused by milling. A typical SL setup consists essentially of four components: (i) a reservoir of UV-sensitive resin in a liquid state, (ii) a non-stationary platform, (ii) a dynamic mirror system, and (iv) a UV laser source. Once the resin layer solidifies, the platform lowers, and another resin layer is deposited on top of the previous layer until the desired 3D structure is achieved. The excess resin is then removed, and the entire 3D structure is cured by UV radiation [20]. Adaptations of SL techniques, such as reducing laser power and improving resolution, have led to the development of new SL techniques, namely micro-stereolithography (1SL), two-photon polymerization (TPP), and digital light processing (DLP). These new SL techniques are more energy-efficient and less time-consuming. The 1SL technique offers extremely high precision because it uses a laser beam with a width of one photon, reducing the laser spot area. Reportedly, this technique produced polypropylene fumarate (PPF) scaffolds with mechanical properties comparable to human cancellous bone [231]. The stereolithography technique along with its processing parameters and additives are illustrated in Figure 15.

### 7.8. Three-Dimensional Printing Methods 

**Figure 16:**
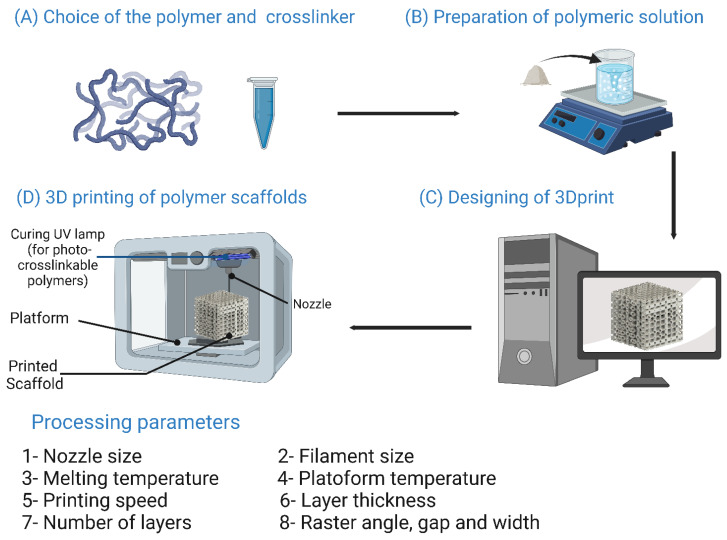
The 3D printing technique and its processing parameters and additives. The image was created using Biorender.

Three-dimensional printing generally occurs at room temperature, where a machine sprays a binder solution onto a layer of powder particles lying horizontally on a platform, causing the powder particles to bond together. Once the first layer is formed, the platform is lowered. Then the next layer is applied and so on in a process common to most additive techniques. A further step is required to remove the remaining powder particles that have not bonded together. This process can be done either directly by molding the desired structure or indirectly by molding a mold for the desired structure [23,232]. Since 3D printing is performed at room temperature, a variety of heat-sensitive biomolecules such as peptides, plasmids, polysaccharides (e.g., alginate and hyaluronan), proteins (e.g., fibrinogen and collagen), and cells that may contribute to new bone formation can be incorporated [19]. Because scaffolds need to be able to carry therapeutic molecules, Tarafder and Bose [233] have reportedly 3D-printed microporous PCL–TCP composite scaffolds to serve as carriers for alendronate (a bisphosphonate used to treat skeletal muscle defects because of its bone resorption inhibitory activity). The scaffolds were used to treat induced distal femoral defects in Sprague Dawley rats for 6 to 10 weeks. Rats implanted with PCL–TCP alendronate-loaded scaffolds showed increased bone formation at 6 weeks compared with rats implanted with TCP and PCL–TCP scaffolds only. Rats implanted with PCL–TCP alendronate-loaded scaffolds formed more compact bone at 6 weeks and more compact bone at 10 weeks. The 3D printing technology allows the fabrication of scaffolds for defect repair that combine different tissues. Sherwood et al. [234] fabricated a two-phase microporous osteochondral composite. The upper layer is composed of D, L-PLGA/PLA for cartilage reconstruction, and the lower layer is composed of L-PLGA/TCP for bone repair. Several parameters can affect the final structure of the resulting framework, such as temperature, printing speed, layer thickness, and filling of the framework. In a study conducted by Baptista et al. [235], the effects of different processing parameters on 3D-printed PLA scaffolds were investigated. It was found that changing the processing parameters has a great impact on the overall morphology of the 3D-printed scaffolds as well as on their mechanical properties. The 3D printing technique along with its processing parameters and additives are illustrated in Figure 16.

Three-dimensional bioprinting (3DBP) is a technique that incorporates cells and biomaterials to create architectures similar to native tissue. The advantages of this novel technology include cost efficiency, scalability, and the possibility of high-precision cell distribution and operation under ambient conditions [24,236]. Hydrogels are primarily utilized for 3DBP [237]. Poldevaart et al. [238] used 3D bioprinting to prepare a biodegradable and non-cytotoxic gelatin-based hydrogel that served as a vehicle for bone morphogenetic protein 2 (BMP-2) microparticles adsorbed on the surface and pores of the hydrogel. Multipotent goat stromal cells and calcium phosphate (CaP) were also incorporated into the hydrogel. The entire bioprinting process took place in a laminar flow cabinet to ensure that the hydrogel was completely sterile. The ability of the cells to undergo osteogenic differentiation and osteogenesis was evaluated both in vitro and in vivo after subcutaneous implantation of the scaffold. Clinically, BMP-2 has been used for postoperative healing of spinal fusions and for healing of tibial defects, but due to the high doses associated with the non-sustained release of BMP-2, some patients began to develop malignancies and excessive growth of bone tissue. Therefore, sustained release of BMP-2 is a promising approach to improve osteogenesis and avoid the complications associated with the use of high doses.

**Figure 17:**
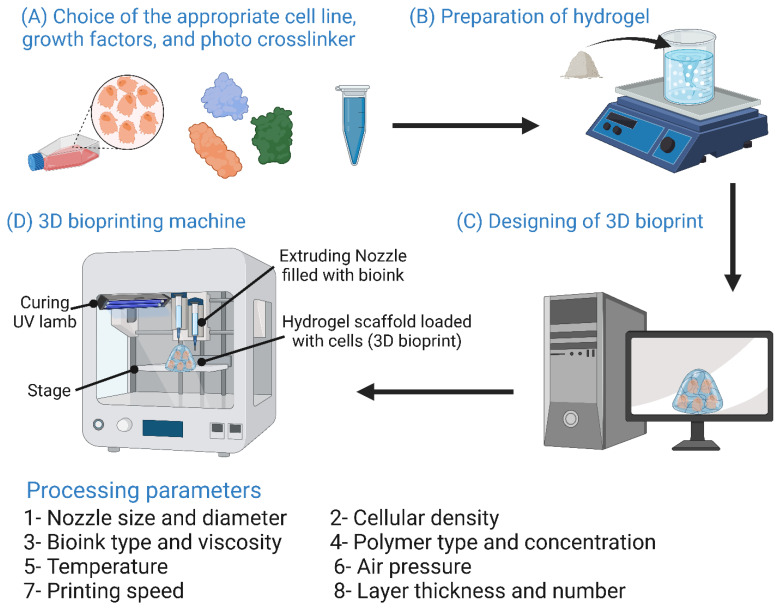
The 3D bioprinting technique and its processing parameters and additives. The image was created using Biorender.

The use of hydrogels for bone tissue engineering is accompanied by several drawbacks, including the inability of hydrogels to withstand large mechanical loads in in vivo studies, insufficient stiffness, a limited critical time of gelation, and low resolution [239]. To compensate for these shortcomings, materials scientists began to focus on the use of materials other than hydrogels. Sawkins et al. [237] fabricated a three-dimensional structure from a thermally reactive PLGA-based material. The results of mechanical testing showed that the structure obtained had mechanical properties similar to cancellous bone, with a tensile strength of 1.22 MPa and a tensile modulus of 57.3 MPa. In addition, the structure was provided with microspheres loaded with the protein lysozyme, which was selected due to its high similarity with BMP-2 in terms of molecular weight and isoelectric point (IEP). The fabricated structure supported sustained release of the protein for 15 days, and the highest measured activity was achieved on day 9. The authors also incorporated human MSCs into the fabricated structure, which showed no cytotoxic effects on the incorporated cells. The 3D bioprinting technique along with its processing parameters and additives are illustrated in Figure 17.

## 8. Challenges Facing Scaffold-Based Bone Tissue Engineering Therapies

The use of scaffolds in BTE still presents some challenges that need to be addressed. First, the appropriate framework-based treatment must be determined, in terms of the choice of material properties, fabrication technique, and whether to use a single-component or multicomponent protocol. Around this core, there are a number of crucial further steps: in vivo and in vitro studies conducted before clinical use, obtaining approval/clearance for clinical trials and conducting those trials, commercializing the scaffold-based therapy developed, and meeting patient expectations. The players in this complex scene include biomaterial scientists who develop and design scaffolds, researchers who perform preclinical studies, surgeons who perform the clinical trials, other members of the clinical practice, companies that turn low-yield laboratory production of scaffolds into large-scale operations to meet market demand, and finally patients with their expectations [19,240].

### 8.1. Identifying the Appropriate BTE Treatment

When it comes to finding the right BTE therapy, orthopedic surgeons must select the BTE treatment that perfectly fits the patient’s clinical condition and reduces the disadvantages of the normally available treatments [241]. Moreover, it is important to have a future perspective of the expected outcome and to consider the expected side effects of this new BTE treatment [240]. A clear understanding of the specificity and severity of the defect, patient clinical data, expected outcome, and BTE treatment protocol is essential to determine the features that materials scientists should incorporate into the fabricated scaffold. When designing a BTE scaffold, materials scientists must closely control the scaffold at three levels: macro, micro, and nano. Control of the scaffold at the macro level is critical to its ability to effectively replace the defective/absent tissue, while organization at the micro level is critical to the scaffold’s osteoconductive and osteoinductive properties, as well as its ability to induce the formation of new blood and bone tissue. Control of the nanoscale features of the scaffold determines its ability to induce protein and cell adhesion, as well as the ability of cells to proliferate and differentiate osteogenically on the surface and through the pores of the scaffold [10].

### 8.2. Multi- vs. Single-Component Therapy

**Figure 18:**
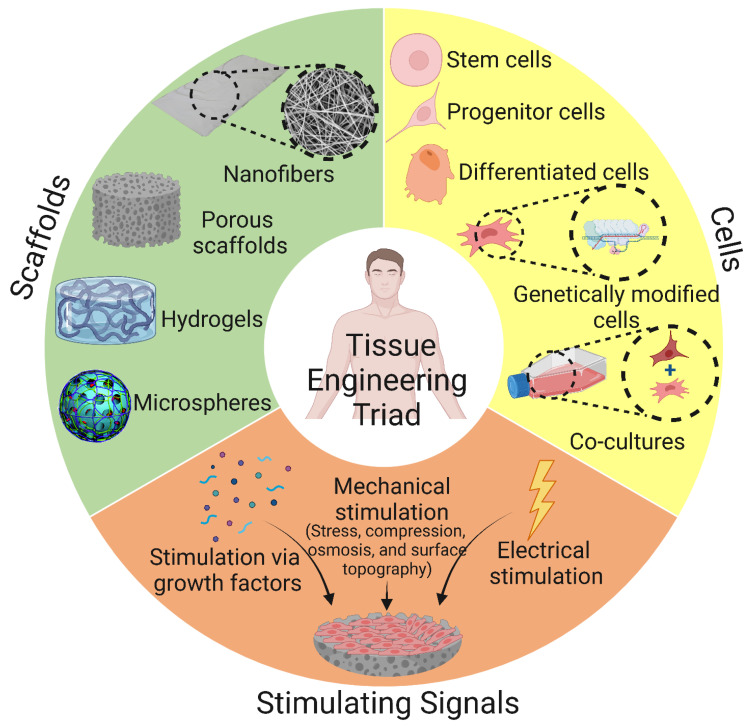
Tissue engineering (TE) triad comprising of three main components: cells, scaffolds, and stimulating signals. The image was created using Biorender.

The TE triad is a three-component system consisting of scaffolds, cells, and stimulatory signals (Figure 18) [242]. Several questions revolve around this triad regarding the choice of individual components and whether to use cell-free scaffolds or scaffolds loaded with cells and/or stimulating factors. These questions include the choice of cell source, the type of cells (differentiated, undifferentiated, unexpanded, ex vivo expanded, progenitor, and genetically modified cells), the number of loaded cells on the scaffold, whether the cells should be expanded as a monolayer or in a bioreactor, whether the cells need to be seeded into the scaffold after fabrication or whether they should be directly bioimprinted with the scaffold, and whether it is better to populate the cells statically or dynamically. To date, choosing the optimal cell source and population has been a challenge [19]. Choosing the appropriate growth factor can also be challenging, as options range from growth factors that induce angiogenesis to osteogenic growth factors to combinations of these two types. There is also the question of how to deliver the growth factors into the scaffold, how to achieve sustained release, and what the optimal dosage is. However, the use of growth factors is associated with several shortcomings, such as the high cost of growth factors, short half-lives, the unstable nature of growth factors, and undesirable complications [19]. For example, the use of BMP-2 in spine and trauma surgery has been reported to be associated with the formation of malignant tumors [243].

### 8.3. Preclinical Investigations

Before BTE treatment paves its way into pilot clinical trials, it must first successfully pass preclinical applications [10]. In vitro investigations are performed either for each component (cells, scaffold, and growth factors) individually or in combination to investigate cellular activities, scaffold cytotoxicity, whether or not the material can elicit an immune response, optimal growth factor dosage and sustained release, and evaluation of cell–biomaterial interactions. Several approaches have been developed to improve in vitro cultivation, including the use of cocultures (in which two or more cell populations are grown with some contact between them) and the use of bioreactors (e.g., perfusion systems, vascular constructs with rotating walls, and mechanically or electromagnetically stimulated cell/scaffold composites) [244,245]. However, in vitro studies cannot accurately predict the response of a living organism to BTE therapy because they are unable to mimic the complexity of living organs and their complex interactions within the living organism [19].

To compensate for the shortcomings of in vitro models, in vivo studies are performed. Performing these studies on small vertebrates such as mice, rats, and rabbits has the advantage that they require less time because of their high bone turnover, the cost of maintaining these animals is relatively low, and they are usually available. However, the significant differences between the skeletal systems of humans and small vertebrates may affect their suitability for in vivo studies. These differences include different loading patterns of bone, negligible intracortical bone healing, absence of Haversian canal systems, open epiphyses at several growth plates (areas of active new bone growth near the bone ends), and finally, the mass of trabecular bone is relatively small compared to the total mass of bone [10]. The superiority of performing in vivo studies on larger vertebrates such as dogs, sheep, pigs, and horses is based on the similarity of their bone properties to those of human bone in terms of microstructure, physiology, and mechanical properties. However, there are some drawbacks that can complicate the use of larger vertebrates for in vivo studies. For example, their slow bone turnover and long lifespan make them time-consuming, and obtaining ethical approval may take a longer time [19].

### 8.4. Clinical Studies Approval and Conduction

Once the novel BTE treatment has successfully passed preclinical testing, clinical trials will begin. However, clinical trials must be conducted on a large number of subjects to validate their efficacy, and the time-consuming process of documenting and approving progress, as well as the need for funding, can also hinder clinical trials. An important issue to consider in clinical trials is the complexity associated with the clinical application of BTE treatment. As a result, the large-scale application of BTE treatments is not yet known [243]. After successful completion of clinical trials, the BTE scaffold must be approved as a biomedical device by organizations such as the Food and Drug Administration (FDA) in the United States and an agency determined by each Member State in Europe. The entire BTE approval process is very time-consuming and can be extremely costly, as it must go through research and development (R&D) processes before being approved by these organizations. To assess the extent to which the proposed BTE treatment is biocompatible, the International Standards Organization (ISO) has established a sequence of standardized tests to evaluate the framework-to-body contact, contact duration, and biological effects exerted [246].

## 9. Future Perspectives

Predicting future advances in the field of BTE is not so simple, as these advances are proceeding rapidly in several directions. Currently, further work and research are needed to fabricate scaffolds capable of developing hierarchically arranged vascularized systems that resemble vascularization in vivo (i.e., vascularized scaffolds). Another important future prospect to consider is the further development of scaffolds with more than one layer for the dual reconstruction of cartilage and bone, as there is currently no marketable BTE treatment for this type of bone-cartilage damage. Finally, and critically, we predict remarkable progress in the production of custom BTE treatments that are specifically tailored to patient needs and expectations. Next, some current efforts to develop vascularized, multilayered, and customized scaffolds will be discussed.

**Figure 19:**
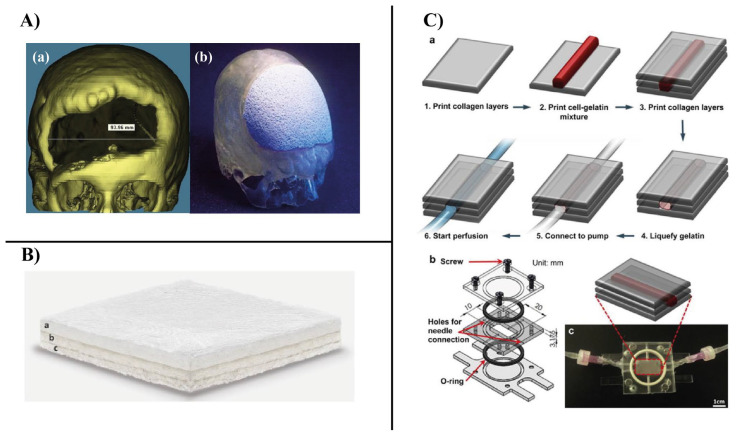
(**A**): (a) A 3D CT scan of a bone cranial defect. The first step to a customized graft is to acquire a patient-specific CT scan image of the defect. (b) Anatomically shaped HA scaffold that perfectly fits the defect in a skull model. A prototype acrylic resin model was fabricated by 1:1 stereolithography, replicating the skull with the defect. A custom HAp prosthesis was then fabricated and accurately refined based on the defect that the resin model exhibited [247]. (**B**): Multilayered composite scaffold with three gradients that replicates the entire articular osteochondral compartment and can initiate osteochondral regeneration. The nanostructured, biomimetic, porous, three-layer gradient composite scaffold mimics (a) the cartilage layer (type I collagen), (b) the tidemark (a combination of type I collagen and non-stoichiometric, magnesium-enriched HA), and (c) the subchondral bone (a mineralized blend of type I collagen and magnesium HA) [248]. (**C**): Method for the preparation of vascular channels using a cell-gelatin mixture. (a) Schematic description of the process. (b) Custom-made flow chamber consisting of three transparent polycarbonate parts and two O-shaped rings for sealing. (c) Image of the flow chamber connected to the perfusion system via side-mounted needles [249].

In a promising attempt to fabricate a custom BTE scaffold, Staffa et al. [247] have reportedly fabricated a three-dimensional custom porous HAp scaffold from bioceramics that reportedly exhibits a high degree of biocompatibility, osteoconductivity, and biomimicry to reconstruct patient skull defects. This 3D custom-made framework has been approved as a biomedical product according to European standards and regulations. To capture the shape and dimensions of the patients’ defects, a 3D model of the defect was developed for each patient using scanning technology CT (Figure 19A(a)). The aforementioned 3D model was used to fabricate a real-size resin-based acrylic model. Then, the implant was fabricated by stereolithography technique using HAp (Figure 19A(b)). After two years of regular follow-up, all patients showed perfect cosmetic appearance. The authors reported only one case of implant rejection in all 51 patients treated.

The urgent need for BTE-based treatment for complicated osteochondral damage led to the development of multilayer scaffolds. A multilayer magnesium/HAp composite scaffold consisting of three different layers was fabricated. The three layers are the cartilage layer, the tidemark, and a subchondral bone layer (Figure 19B). In vitro studies showed that the implanted mesenchymal stem cells were able to differentiate into chondrocytes and bone cells, resulting in two distinct layers of cartilage and bone. Using RP technologies, it was possible to create in vitro networks of hierarchically arranged vessels similar to those in vivo [248]. Lee et al. [249] reportedly fabricated an innovative flow chamber by placing a mixture of human umbilical vein endothelial cells (HUVECs) on top of a collagen-based layer followed by additional collagen layers. To ensure that the inner walls of the chamber were completely covered with cells, the chamber was turned upside down every 10 to 15 min. To remove the gelatin, the medium was carefully pumped into the chamber at a rate of 0.3 mL/min. As reported by the authors, HUVECs were able to adhere to the inner walls of the chamber, resulting in a vascularized structure with high biomimicry (Figure 19C).

## 10. Conclusions

Polymeric materials have proven to be extremely advantageous in the fabrication of BTE scaffolds. Scaffolds fabricated with natural polymers have been shown to exhibit exceptional biocompatibility, biodegradability, osteoconductivity, and osteoinductivity. However, their inferior mechanical properties led them to be combined with synthetic polymers to improve their mechanical properties. Moreover, polymeric composites blended with bioactive ceramics and bioactive molecules have been shown to have remarkable osteoinductive and osteoconductive effects, leading to an improvement in their bone healing efficiency as well as improving their immunomodulatory effects, leading to the alleviation of the inflammatory response accompanied by their implantation. Conventional BTE scaffold fabrication techniques have resulted in BTE limitations and need to be replaced by newer and less cytotoxic rapid prototyping techniques that compensate for the limitations associated with conventional techniques. Indeed, the use of polymeric scaffolds in BTE will contribute significantly to reducing the long waiting lists for bone grafting. However, the use of scaffold materials in biomedical applications currently faces many challenges. Among the aforementioned challenges is the selection of the correct and most appropriate TE treatment for the patient, the time-consuming and costly preclinical and clinical investigations and obtaining approval to conduct clinical trials, and the lengthy and complicated procedures required to obtain approval of the novel TE treatment as a biomedical product, and even these procedures and regulations may vary from country to country, hindering the possibility of worldwide use of the novel BTE treatment.

## Figures and Tables

**Table 1:** Examples of natural polymers used as biomaterials for the production of BTE scaffolds along with their advantages and disadvantages.

Polymer	Advantages	Disadvantages	Refs.
Protein-based natural polymers			
Collagen	- Biocompatibility - Biodegradability - Fiber-like nature - Biomimicry - Low antigenicity - Self-renewing ability - High ability to promote cellular adhesion - Bio-functionality	- Inferior mechanical properties - Inferior chemical stability - Relatively low melting point - Hard to process - Hard to manipulate the rate of degradation - Nanofibers tend to fuse together in aqueous environments - High costs to be produced via recombinant approaches	[100,101,102]
Gelatin	- Biocompatibility - Biodegradability - Anti-thrombotic effects - Presence of cell recognition sites - Low antigenicity - Easy to be molded into different shapes with different dimensions (injectable hydrogels)	- Low chemical stability - Low mechanical properties - High brittleness - The necessity for chemical crosslinking	[48,103]
Silk Fibroin	- Biocompatibility - Biodegradability - Elevated thermal stability - Remarkable mechanical properties - High tensile strength	- Inadequate supply (as its production is restricted to some moths and spiders) - Relatively high brittleness - Requirement for additional steps to remove other contaminants	[104,105]
Hyaluronic acid	- Biocompatibility - Biodegradability - Highly viscoelastic - Remarkable solubility in water - Excellent biomimicry as it is a component of natural ECM and has a high resemblance to GAGs - Ease of manufacture in large quantities via microbial action - Ease of functionalization	- Hard to be electrospun into nanofibers due to its high viscosity and surface tension - Inferior mechanical competence - High costs of preservation as it requires to be stored in a cryo-freezer	[104,106,107]
Peptides	- Biocompatibility - Biodegradability	- Inferior mechanical properties	[108,109]
Keratin	- Biocompatibility - Biodegradability	- Inferior mechanical properties	[89,105,110]
Fibrin	- Biocompatibility - Biodegradability - Excellent cell-to-matrix interaction	- Inferior mechanical properties - Inferior mechanical and thermal stability - Relatively low osseointegration - Relatively quick degradation in vivo	[48,111]
Heparin	- Biocompatibility - Biodegradability - Excellent choice for growth factor loading	- Decreased rate of cellular growth	[112]
Polysaccharide-based natural polymers			
Chitosan	- Biocompatibility - Biodegradability - Low antigenicity - Low cytotoxicity - Ease of extraction and low costs of manufacture - Remarkable antimicrobial activity - Capable of self-renewal - High capability to load negatively charged molecules due to its positively charged surface	- Hard to be electrospun into nanofibers - Low osteoconductivity - Inferior mechanical properties - Inferior chemical and thermal stability	[48,104,113]
Alginate	- Biocompatibility - Biodegradability - Can easily form gels - Relatively easy to functionalize - Easily crosslinked and can form injectable gels - Can withstand acidic conditions - Tunable properties which are dependent on the ratio of its two monomers	- Inferior mechanical properties - Leaching of loaded bioactive molecules and drugs - Unpredictable degradation patterns - Hard to sterile and difficult to handle	[104,105]
Cellulose	- Highly abundant and readily available - Biocompatibility - Low costs of preparation and extraction - Porous structure - Ease of conversion to its derivatives	- Prolonged self-renewal - Low biodegradability in vivo	[106,114]
Starch	- Biodegradability - High abundance - Capable of self-renewal	- Relatively high brittleness - Difficult to process - Notable semi-crystalline regions of starch during processing	[106,115]
Agar	- Biodegradability - Biocompatibility	− Difficult to process and extract	[48,116]
Dextran	- Biodegradability - Biocompatibilityz - The availability of several derivatives with a variety of molecular weights	- High cost of isolation - Overhydration - Suspected to being coagulated - High risk of anaphylaxis development	[48,117]

## Data Availability

Not applicable.
